# Listening in a noisy world: The impact of acoustic cues and background music on speech perception in autism

**DOI:** 10.1177/13623613251376484

**Published:** 2025-10-14

**Authors:** Jiayin Li, Maleeha Sujawal, Zivile Bernotaite, Ian Cunnings, Fang Liu

**Affiliations:** University of Reading, UK

**Keywords:** acoustic cue, autism, background music, speech-in-noise processing

## Abstract

Recognising speech in noise involves focusing on a target speaker while filtering out competing voices and sounds. Acoustic cues, such as vocal characteristics and spatial location, help differentiate between speakers. However, autistic individuals may process these cues differently, making it more challenging for them to perceive speech in such conditions. This study investigated how autistic individuals use acoustic cues to follow a target speaker and whether background music increases processing demands. Thirty-six autistic and 36 non-autistic participants, recruited in the United Kingdom, identified information from a target speaker while ignoring a competing speaker and background music. The competing speaker’s gender and location either matched or differed from the target. The autistic group exhibited lower mean accuracy across cue conditions, indicating general challenges in recognising speech in noise. Trial-level analyses revealed that while both groups showed accuracy improvements over time without acoustic cues, the autistic group demonstrated smaller gains, suggesting greater difficulty in tracking the target speaker without distinct acoustic features. Background music did not disproportionately affect autistic participants but had a greater impact on those with stronger local processing tendencies. Using a naturalistic paradigm mimicking real-life scenarios, this study provides insights into speech-in-noise processing in autism, informing strategies to support speech perception in complex environments.

**Lay abstract:**

This study examined how autistic and non-autistic adults understand speech when other voices or music were playing in the background. Participants focused on one main speaker while another voice played simultaneously. Sometimes, the second voice differed from the main one in gender or where the sound was coming from. These differences made it easier to tell the voices apart and understand what the main speaker was saying. Both autistic and non-autistic participants did better when these differences were present. But autistic individuals struggled more when the two voices were the same gender and came from the same location. Background music also made it harder to understand speech for everyone, but it especially affected autistic participants who tended to focus more on small details. These findings help us understand how autistic individuals process speech in noisy environments and could lead to better ways to support communication.

## Introduction

Listening in a cocktail-party environment, where multiple speakers and background sounds overlap, places significant demands on both bottom-up and top-down processing mechanisms (Bronkhorst, 2000). Bottom-up processes enable the detection of acoustic differences between competing auditory streams, while top-down mechanisms, including selective attention and cognitive control, guide the selection of relevant auditory information and help suppress irrelevant distractions. Together, these mechanisms integrate acoustic information into cohesive auditory objects, facilitating effective speech recognition in noise (Başkent & Gaudrain, 2016; Shinn-Cunningham & Best, 2008).

Autistic individuals often face challenges with speech-in-noise (SiN) recognition, due to differences in auditory processing and cognition (O’Connor, 2012; Ruiz Callejo & Boets, 2023). These difficulties may exacerbate social communication difficulties (American Psychiatric Association, 2013), as social interaction often occurs in complex listening environments. Behaviourally, autistic individuals struggle to use brief reductions in noise intensity to recognise target speech (Alcántara et al., 2004; Groen et al., 2009; Schelinski & Von Kriegstein, 2020). Electrophysiological studies further report reduced spatial attention and diminished frequency discrimination in the presence of competing auditory streams (Lepistö et al., 2009; Teder-Sälejärvi et al., 2005). Moreover, attenuated neural encoding of vowels predicts difficulties in word-in-noise recognition, suggesting the effect of early acoustic disruptions on SiN comprehension (Fadeev et al., 2024). Collectively, these results indicate autistic individuals’ difficulties in extracting and integrating acoustic information during SiN processing.

In multi-speaker environments, differences in spatial location and vocal characteristics can be used to segregate overlapping speech and follow the target speaker (Culling et al., 2003; Darwin et al., 2003; Shinn-Cunningham et al., 2017). However, how autistic individuals use these cues remains unclear, despite frequent reports of day-to-day difficulties in managing competing voices (Bendo et al., 2024). Emmons et al. (2022) addressed this by examining whether participants could use gender and location cues to direct attention between competing speakers. Non-autistic participants performed at ceiling with either cue alone, whereas autistic participants showed better performance only when both cues were available, indicating a greater reliance on multiple cues. Lau et al. (2023) found that lower SiN recognition in a three-speaker scenario was associated with lower intelligence quotient (IQ), but they did not directly examine how acoustic cues affected performance.

Another socially relevant yet often overlooked challenge is the interference of background music with speech recognition (Brown & Bidelman, 2022; Russo & Pichora-Fuller, 2008; Shi & Law, 2010). Autistic individuals often prefer music over speech, likely due to its structured, predictable and emotionally resonant qualities, in contrast to the nuanced variability and social complexity of spoken language (Allen et al., 2009; Kuhl et al., 2005). Neural evidence suggests that autistic children show stronger brain responses to music than to speech or environmental noise, indicating heightened sensitivity to musical input (Molnar-Szakacs & Heaton, 2012). Many autistic individuals also demonstrate enhanced musical abilities including superior pitch perception and melodic memory despite well-documented difficulties with speech and language processing (Heaton et al., 2008; O’Connor, 2012; Ouimet et al., 2012). These findings suggest that music is particularly salient for autistic individuals and may draw greater perceptual and cognitive resources when presented concurrently with speech, potentially making it a more disruptive distractor during speech processing.

This study addressed two key research questions:

1. Do autistic participants benefit from acoustic cues in resolving SiN challenges in a two-speaker scenario?
2. Does background music impose greater processing demands on autistic listeners compared to non-autistic listeners?

Participants were asked to identify speech from a target speaker presented simultaneously with a distractor speaker and instrumental background music. The spatial location and gender of the distractor were systematically manipulated, creating four conditions: no-cue, gender-cue, location-cue and both-cues conditions. Importantly, the relative loudness of the speech compared to the background noise (i.e. signal-to-noise ratio, SNR) was kept fixed throughout the experiment. This contrasts with previous studies that adaptively varied SNR to estimate speech detection thresholds (Alcántara et al., 2004; Groen et al., 2009). We maintained a fixed SNR to account for auditory hypersensitivity commonly reported in autism (Williams et al., 2021), as the gradual increase in noise used in adaptive procedures could cause sensory discomfort and, in turn, confound measures of speech perception (Danesh et al., 2021; Khalfa et al., 2004).

Beyond group comparisons of mean accuracy, we used Generalised Additive Mixed Models (GAMMs; Wood, 2011, 2017) to analyse accuracy trajectories over trials across cue conditions and groups. By tracking changes in performance over time, this approach captured non-linear patterns that may reflect improvement, attentional shifts or fatigue during the task. Building on prior evidence suggesting less efficient use of auditory cues as well as general SiN-processing difficulties in autism, we expected autistic participants to show lower overall accuracy across conditions and slower improvement over trials, particularly in conditions with fewer available cues. This would reflect greater difficulty in tracking the target speaker when salient acoustic distinctions are absent. We also hypothesised that background music would interfere more with speech recognition in the autistic group, based on previous findings that music is often more salient, emotionally engaging and perceptually preferred over speech in autistic individuals (Heaton et al., 2008; Molnar-Szakacs & Heaton, 2012; Ouimet et al., 2012).

Successful performance on this task requires participants to recognise and effectively utilise acoustic cues to segregate a target speaker from competing streams. This involves perceiving individual features and integrating them into a coherent auditory object over time, a process may be particularly demanding for autistic individuals. According to weak central coherence (WCC) theory, autistic individuals show a cognitive bias towards local over global information, which may affect their ability to combine multiple auditory cues across time and sources (Happé & Frith, 2006). Predictive coding accounts offer a complementary explanation, suggesting reduced reliance on top-down predictions, which may limit their ability to anticipate and filter relevant speech in noisy or unpredictable contexts (Van De Cruys et al., 2014). Neurobiological accounts further propose that reduced functional connectivity and lower signal complexity may compromise the integration of acoustic information into a coherent target stream (Belmonte et al., 2004; Just et al., 2012).

Accordingly, if these perceptual tendencies constrain acoustic integration in the current task, other cognitive mechanisms such as working memory and reasoning may compensate to support task performance. Conversely, if autistic individuals demonstrate heightened sensitivity to local, low-level features, as proposed by the Enhanced Perceptual Functioning account (Mottron et al., 2006), we might expect stronger associations between pitch discrimination ability and task accuracy, reflecting a reliance on fine-grained auditory detail. Therefore, to assess whether theoretically motivated individual differences contribute to SiN performance, we conducted a correlational analysis examining the associations between task accuracy and non-verbal IQ, working memory, pitch discrimination and local-to-global processing style. This approach allowed us to evaluate whether these cognitive factors support cue-based listening and whether different mechanisms may be involved across groups.

## Methods

### Participants

A power analysis determined a target sample size of 70 participants (35 per group), providing nearly 80% power to detect most effects of interest (see Supplementary Material for details). Ultimately, we recruited 36 autistic and 36 non-autistic native English speakers aged 16 to 47. All participants had normal or corrected-to-normal vision, no colour blindness and normal pure-tone hearing levels at 0.5, 1, 2 and 4 kHz. Both groups had no current speech, language or communication needs. Clinical diagnoses were confirmed for all autistic participants, while non-autistic participants had no personal or family history of autism and scored below 32 on the Autism Spectrum Quotient (AQ; Baron-Cohen et al., 2001).

To account for potential factors influencing SiN processing, we assessed cognitive and auditory abilities as well as musical training background (see Table 1 for participant demographics). Cognitive measures included non-verbal IQ (Raven’s Standard Progressive Matrices; Raven & Court, 1998), receptive vocabulary (Receptive One-Word Picture Vocabulary Test–Fourth Edition (ROWPVT-4); Martin & Brownell, 2011) and verbal short-term memory (digit span task; Wechsler et al., 2003). Cognitive processing style was assessed using Navon’s paradigm (Navon, 1977) in which participants responded to composite letters in congruent (e.g. a large H composed of small Hs) or incongruent (e.g. a large H composed of small Ss) configurations. Two metrics were derived: global advantage (reaction time (RT) difference for global vs local judgements on congruent trials, indicating a bias towards global processing) and local-to-global interference (RT difference for global judgements between congruent and incongruent trials, reflecting difficulty prioritising global over conflicting local information). Navon scores were based on 62 participants, as 10 (5 autistic, 5 non-autistic) did not complete the task.

**Table 1.** Characteristics of the autistic (*n* = 36) and non-autistic groups (*n* = 36).

Variables	Autistic mean (*SD*)	Non-autistic mean (*SD*)	*W*	*p*	Rank-biserial correlation
Gender (Female:Male:Others)	22:12:2	28:7:1			
Age	23.29 (5.60)	23.54 (5.99)	641.0	0.942	–0.01
Autistic traits (AQ)	37.89 (7.20)	16.08 (7.53)	1258	< 0.001	0.94
Musical training years	5.01 (6.67)	5.67 (7.67)	608.5	0.647	–0.06
Non-verbal reasoning (RSPM raw score)	54.00 (3.68)	54.08 (3.26)	669.5	0.810	–0.03
Non-verbal reasoning (RSPM percentile)	51.94 (25.62)	51.5 (26.43)	661.5	0.879	0.02
Receptive vocabulary (ROWPVT-4 raw score)	166.56 (10.77)	167.50 (10.77)	633.0	0.870	–0.02
Receptive vocabulary (ROWPVT-4 standard score)	111.25 (16.27)	112.47 (14.15)	622.0	0.774	–0.04
Digit span	7.03 (1.48)	7.25 (1.18)	562.0	0.322	–0.13
Pitch threshold	0.22 (0.10)	0.33 (0.47)	644.5	0.973	–0.01
Global advantage	107.87 (103.21)	102.88 (43.99)	467.0	0.850	0.03
Local-to-global interference	20.41 (26.88)	15.21 (76.89)	652.0	0.016	0.36

AQ: Autism Spectrum Quotient; RSPM: Raven’s Standard Progressive Matrices; ROWPVT-4: Receptive One-Word Picture Vocabulary Test, 4th edition.

Auditory abilities were evaluated using a pitch direction discrimination task (Liu et al., 2010), where thresholds were determined through a ‘two down, one up’ adaptive staircase method. Musical training background was measured as self-reported years of formal instrumental and vocal training, with total training years used as the primary metric (Pfordresher & Halpern, 2013).

Wilcoxon rank-sum tests revealed no significant differences between the autistic and non-autistic groups on key demographic and cognitive variables. Autistic participants scored significantly higher on the AQ. They also exhibited higher local-to-global interference scores on the Navon task, suggesting increased difficulty in prioritising global over local information.

This study received ethical approval from the University Research Ethics Committee. All participants provided written informed consent before participating and received financial compensation or course credits for their involvement.

### Stimuli and apparatus

The target and distractor speech stimuli were sourced from the Children’s Coordinate Response Measure corpus (Messaoud-Galusi et al., 2011), recorded by three Southern Standard British English speakers. Each sentence followed the structure: ‘Show the ANIMAL (dog/cat) where the COLOUR (black/blue/green/pink/red/white) NUMBER (1/2/3/4/5/6/8/9) is’. The number ‘7’ was excluded due to its two-syllable pronunciation, making it easier to distinguish from the other numbers. To direct attention, the callsign ‘dog’ was always used for the target speaker, while distractor speakers used the callsign ‘cat’.

**Figure 1.**
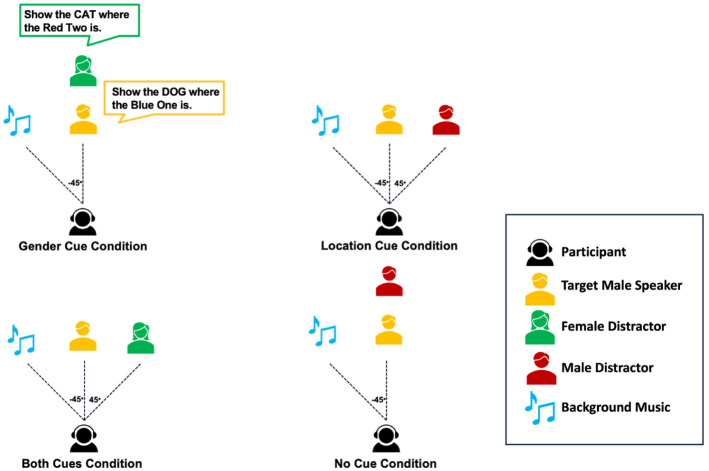
Schematic representation of design. The figure illustrates a single configuration of distractor and music locations; in the actual experiment, these locations vary dynamically across trials, with the music positioned symmetrically opposite the distractor.

Figure 1 provides an overview of the experimental design. Acoustic cues were manipulated by varying the distractor’s gender (matching or differing from the target) and spatial location (co-located or separated from the target), resulting in four conditions: (1) no-cue: matched gender, co-located position; (2) gender-cue: mismatched gender, co-located position; (3) location-cue: matched gender, separated positions; (4) both-cues: mismatched gender, separated positions. Spatial separation was achieved using binaural head-related transfer functions, which simulate realistic spatial positioning over headphones (Wenzel et al., 1993; Wightman & Kistler, 1989). In our setup, the target speaker was fixed at 0° azimuth, while distractors were either co-located or positioned at –45° (left) or +45° (right). This was experienced by participants as the distractor voice shifting towards the left or right ear, making it perceptually distinct from the centrally presented target.

To evaluate the effect of background music, half of the trials included peaceful instrumental music spatially mirrored to the distractor speaker’s location. Music stimuli were derived from a validated set of film music excerpts developed to reflect distinct emotional qualities (Eerola & Vuoskoski, 2011). To minimise emotional or semantic interference while maintaining ecological validity, we chose excerpts characterised by high valence and low arousal. These pieces feature a slow tempo, smooth dynamics and soft timbres (see Supplementary Material for details). Each excerpt was taken from the middle section of the original track, avoiding the beginning and end where dynamic or structural changes are more likely to occur. This ensured consistent volume and texture throughout the listening period.

The target speech was played at 55 dB SPL through headphones, with background music (when present) adjusted to a 0 dB SNR, ensuring equal intensity between the music and target speech.

To balance task difficulty with performance feasibility and ecological validity, we included two SNR levels (−9 dB and −3 dB) determined through pilot testing (see Supplementary Material for details). Neurotypical participants achieved 60%–70% accuracy at −9 dB across conditions, indicating substantial but manageable difficulty. The −3 dB level was included to ensure the task remained accessible to autistic individuals who may experience heightened sensitivity to sound intensity, potentially leading to discomfort or disengagement in more challenging conditions (Danesh et al., 2021; Khalfa et al., 2004). These effects may arise at a sensory processing level, independent of auditory segregation ability. Including only −9 dB trials could risk floor effects or excessive sensory load in the autistic group. The −3 dB level was, therefore, added to reduce the overall sensory burden.

Notably, SNR was not analysed as an experimental variable, and participants were not informed of the level changes during the experiment. Instead, the two SNR levels were randomly intermixed within blocks to introduce a naturalistic and unpredictable listening environment. This approach prioritised participant accessibility, ecological validity and engagement, while avoiding potential confounds related to sensory sensitivity and ensuring that the task remained feasible and realistic across both groups.

### Procedure

The experiment was implemented and presented using PsychoPy (version 2022.2.2; Peirce et al., 2019). On each trial, participants listened to either two or three simultaneous auditory streams delivered via headphones including a target speaker, a distractor speaker and, in some trials, background music. Participants were instructed to focus on the target speaker, identified by the callsign ‘dog’ and report the associated colour-number combination while ignoring the distractor speaker using the callsign ‘cat’. Following the auditory stimulus, participants were shown an on-screen response grid containing all possible colour-number combinations (see Supplementary Material) and responded by clicking on the corresponding coloured number box as quickly and accurately as possible using a computer mouse. A correct response required selecting both the correct colour and number. The target and distractor speakers never shared the same callsign, colour or number. Before the experiment, participants were informed that music might be present in some trials but were not given pre-trial cues about its presence.

The experiment consisted of 288 trials, combining 48 unique colour–number pairs (six colours × eight numbers), two distractor genders (male, female) and three spatial locations (co-located, left, right). For analysis, performance in left and right distractor conditions was averaged to represent conditions with location cues. Trials were randomly presented across six blocks, with breaks between blocks to minimise fatigue. Conditions were mixed within each block to prevent participants from anticipating the presence of background music or specific acoustic cues. No prior information was given about the target speaker’s gender or location. Before the main experiment, participants completed a training session consisting of eight trials with feedback to confirm their understanding of the task and the audibility of the target sentences.

### Statistical analysis

All analyses were conducted in R (version 4.1.2, Posit Team, 2022).

#### Linear mixed-effects models

To examine the three-way interaction between acoustic cues, background music and group, linear mixed-effects models (LMMs) were constructed using the lme4 package (Bates et al., 2015). Accuracy was analysed using generalised LMMs (GLMMs) with the BOBYQA optimiser. Reaction times (RTs) for correct responses were analysed using LMMs, with RTs log-transformed to correct for positive skewness. Outliers exceeding three standard deviations from each participant’s mean RTs across conditions (< 2% of the data) were excluded.

Gender-cue and location-cue conditions were averaged into a single one-cue condition. Helmert coding was applied to compare cue conditions: (1) Cue1 (No cue vs Any cue): No cue = 2/3; One cue = –1/3; Both cues = –1/3; (2) Cue2 (One cue vs Both cues): No cue = 0; One cue = 1/2; Both cues = –1/2. ‘Any cues’ refers to trials where at least one cue was present, encompassing both the one-cue and both-cues conditions. Fixed effects in the models included group (autistic = 1/2, non-autistic = −1/2), background music (without music = 1/2, with music = −1/2), acoustic cue (Cue1, Cue2), and their interactions.

Models were first fitted with maximal random effects structures, including by-participant and by-item random intercepts and slopes for all relevant fixed effects (Barr, 2013). Due to convergence issues, the structure was simplified in stages: first by removing correlations among random effects, then by removing random intercepts and finally by adopting a forward selection approach. This involved starting with a model containing only random intercepts and incrementally adding random slopes, retaining only those that significantly improved model fit based on likelihood ratio tests. The final model reflected the most complex convergent structure. Fixed effects and interactions were assessed via likelihood ratio tests by comparing the final model to nested models with specific effects removed. Significant interactions were explored through simple effects analyses on subsetted data. All follow-up models used the most complex convergent structure shared across subsets. Bonferroni correction was applied.

#### Generalised additive mixed model

To investigate the effects of group and cue condition on accuracy over time, we conducted a GAMM analysis using the mgcv (Wood, 2011, 2017) and itsadug packages (van Rij et al., 2015). Tensor function plots were generated to visualise interaction effects, identifying time windows of significant differences across group and condition (focusing on the no-cue and both-cues conditions). The SNR levels were randomly presented across conditions, which could potentially confound the Group × Condition interaction effect across trials. To address this, we constructed separate GAMMs for each SNR (see details in Supplementary Material).

#### Pearson correlation

A Pearson correlation analysis was conducted to examine the relationship between individual cognitive factors and task performance. To maintain the integrity of our hypothesis-driven analysis, we did not apply multiple corrections, as this could obscure meaningful effects. This approach is consistent with recent methodological guidance suggesting that such corrections are not always necessary when testing a small number of a priori hypotheses and when no omnibus null hypothesis is being evaluated (García-Pérez, 2023). Factors included non-verbal IQ, working memory, musical training and pitch-processing ability, all previously linked to SiN processing (Gordon-Salant & Cole, 2016; Heinrich, 2021). Navon task scores were also examined to assess global–local processing style, given evidence of local processing bias in autism (Happé & Frith, 2006), which could influence the ability to integrate auditory information. Receptive vocabulary was not included, as our use of consistent sentence structures minimised lexical demands.

We examined three performance measures: overall accuracy, accuracy in the no-cue condition (the most difficult) and the background music effect (the accuracy difference between without- and with-music conditions). To normalise percentage accuracy scores, the rationalised arcsine transformation was applied before analysis (Studebaker, 1985).

### Community involvement

There was no community involvement in this study.

## Results

### Linear mixed-effects models

#### Accuracy

**Figure 2.**
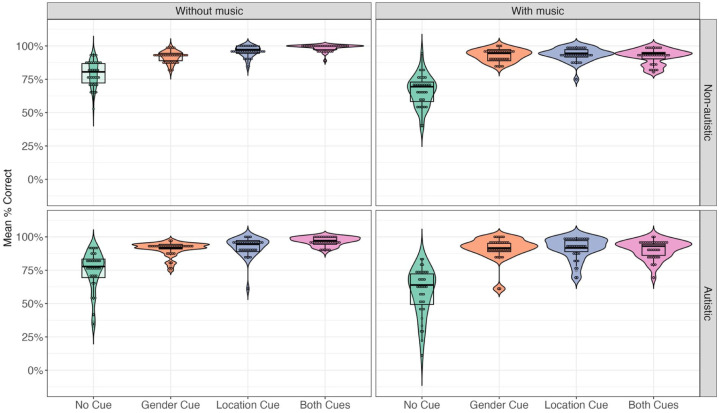
Mean accuracy rate across groups and conditions.

Figure 2 displays the mean accuracy across cue conditions and groups, while Table 2 summarises the model results. A significant main effect of group revealed that autistic participants (*M* = 85.9%, *SD* = 34.8%) exhibited lower accuracy than their non-autistic counterparts (*M* = 88.9%, *SD* = 31.4%). Significant main effects were found for both acoustic cue contrasts. Accuracy was lower in the no-cue condition than in trials with at least one cue. In addition, accuracy in the one-cue condition (gender: *M* = 91.7%, *SD* = 27.5%; location: *M* = 93.4%, *SD* = 24.9%) was lower than in the both-cues condition (*M* = 94.6%, *SD* = 22.5%).

**Table 2.** Results of the GLMM for behavioural accuracy.

Fixed effects	β	*SE*	*z*	OR	95% CI	χ^2^	*p*
(Intercept)	2.88	0.13	22.94	—	—	—	—
Group	–0.41	0.18	–2.26	0.66	[0.46, 0.95]	4.88	**0.027**
Music	0.94	0.18	5.20	2.56	[1.80, 3.64]	25.78	**<** **0.001**
Cue1	–2.56	0.20	–12.77	0.08	[0.05, 0.11]	135.31	**<** **0.001**
Cue2	–0.75	0.22	–3.41	0.47	[0.31, 0.73]	11.44	**<** **0.001**
Group × Cue1	0.05	0.16	0.34	1.05	[0.77, 1.44]	0.11	0.737
Group × Cue2	0.27	0.19	1.42	1.31	[0.90, 1.89]	1.86	0.172
Music × Cue1	–0.01	0.38	–0.04	0.99	[0.47, 2.09]	0.00	1.000
Music × Cue2	–1.48	0.44	–3.36	0.23	[0.10, 0.54]	10.99	**<** 0.001
Group × Music	–0.24	0.14	–1.81	0.78	[0.60, 1.02]	3.06	0.080
Group × Music × Cue1	0.64	0.24	2.69	1.90	[1.19, 3.05]	6.87	**0.008**
Group × Music × Cue2	0.79	0.38	2.09	2.20	[1.05, 4.62]	4.06	**0.044**

GLMM: generalised linear mixed-effects model; OR: odds ratio.

Odds ratios are obtained by exponentiating the model’s log-odds (β) coefficients. 95% confidence intervals (CIs) are similarly derived by exponentiating the CIs of the log-odds estimates. Significant *p*-values are presented in bold.

There was a significant main effect of background music. Accuracy was lower in the with-music condition (*M* *=* 84.6%, *SD* = 36.1%) compared to the without-music condition (*M* = 90.1%, *SD* = 29.9%). We also observed significant three-way interactions between group, music and each cue contrast. To follow up, we conducted separate analyses for each interaction (see Supplementary Material for full results). The only significant comparison emerged in the non-autistic group, where accuracy in the both-cues condition was significantly lower with background music than without it, χ²(1) = 23.64, *p* < 0.001, OR = 11.78, 95% CI = [4.08, 34.00]. No other comparisons yielded significant effects (all *p*-values > 0.09).

#### Reaction times

**Figure 3.**
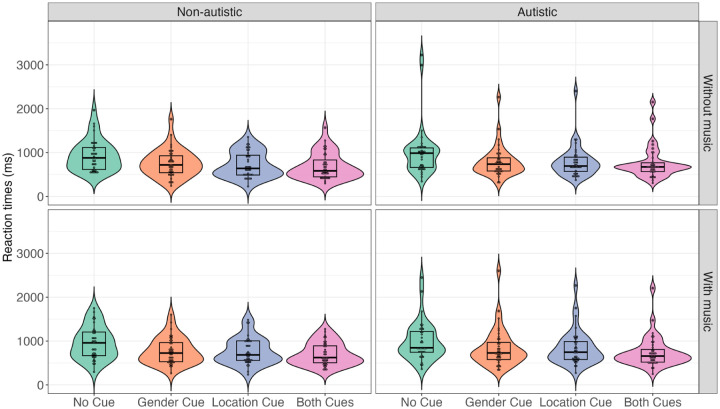
Mean RTs across groups and conditions. RT: reaction time.

Figure 3 presents the mean RTs across conditions and groups. The final model included fixed effects and by-item and by-participant random intercepts. Consistent with the accuracy results, a significant main effect of acoustic cues was observed. Longer RTs were required for accurate responses in the no-cue condition (*M* = 939.80, *SD* = 819.73) compared to the both-cues condition (*M* = 722.34, *SD* = 610.13) and the one-cue condition (gender-cue: *M* = 782.39, *SD* = 637.48; location-cue: *M* = 773.87, *SD* = 637.07). However, no significant main effects of music or group were found. In addition, no significant interactions between these factors were observed (see Table 3).

**Table 3.** Results of the LMM for reaction times (RTs) of accurate responses.

Fixed effects	β	*SE*	*t*	Exp(β)	95% CI	χ^2^	*p*
(Intercept)	6.41	0.05	126.76	—	—	—	—
Group	0.06	0.10	0.66	1.07	[0.88,1.30]	0.43	0.512
Music	–0.03	0.02	–1.44	0.97	[0.92,1.01]	2.07	0.150
Cue1	0.27	0.03	9.77	1.31	[1.24,1.38]	84.21	**<** **0.001**
Cue2	0.10	0.03	3.48	1.10	[1.04,1.16]	11.88	**<** **0.001**
Group × Cue1	0.00	0.03	0.17	1.00	[0.95,1.06]	0.03	0.864
Group × Cue2	0.00	0.03	0.06	1.00	[0.95,1.05]	0.00	0.950
Music × Cue1	0.00	0.05	0.01	1.00	[0.90,1.11]	0.00	1.000
Music × Cue2	–0.03	0.05	–0.60	0.97	[0.87,1.08]	0.36	0.547
Group × Music	0.04	0.02	1.92	1.05	[1.00,1.09]	3.52	0.061
Group × Music × Cue1	0.00	0.05	–0.02	1.00	[0.90,1.11]	0.00	0.988
Group × Music × Cue2	-0.06	0.05	–1.15	0.94	[0.85,1.04]	1.31	0.252

Exp(β) values are obtained by exponentiating the fixed-effect coefficients from the linear mixed-effects model predicting log-transformed response times. The resulting values reflect multiplicative effects on raw response times, where values greater than 1 indicate longer response times and values less than 1 indicate shorter response times relative to the reference level. The accompanying 95% confidence intervals (CIs) are derived by exponentiating the intervals for the log-scale estimates. Significant *p*-values are presented in bold.

### Generalised additive mixed models

**Figure 4.**
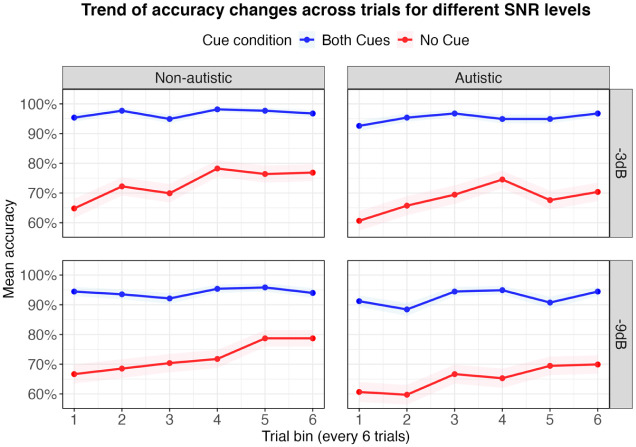
The trend of mean accuracy changes across trial bins (every six trials) for different SNR levels across group and condition with the shaded area indicating the 95% confidence interval. SNR: signal-to-noise ratio.

To investigate how SiN performance changed over time, we used GAMMs to model trial-level accuracy trajectories across cue conditions (no-cue vs both-cues) and groups (autistic vs non-autistic) for each SNR level. Each model included parametric effects for group and cue condition as well as smooth terms to capture time-varying trends within each group–condition combination. Participant-specific smooth terms were also included to account for individual variability (see Table 4). Figure 4 illustrates accuracy trends across trials (in bins of six) for each group and cue condition. As can be seen, both groups performed at ceiling in the both-cues condition with little change across trials. In contrast, in the no-cue condition, both groups showed improvements over time – particularly the non-autistic group, whose performance steadily increased across trials.

**Table 4.** Summary of GAMMs for accuracy by Group and Cue at each SNR level.

	–3 dB SNR				–9 dB SNR			
Parametric coefficients	β	*SE*	*z*	*p*	β	*SE*	*z*	*p*
(Intercept)	3.10	0.16	19.53	< **0.001**	2.65	0.15	18.00	< **0.001**
NAS.Both cues	0.36	0.24	1.48	0.138	0.21	0.21	0.99	0.322
AS.No cue	–2.32	0.15	–15.81	< **0.001**	–2.00	0.13	–15.93	< **0.001**
NAS.No cue	–2.08	0.19	–10.87	< **0.001**	–1.65	0.19	–8.79	< **0.001**
Smooth terms	edf	Ref.df	χ²	*p*	edf	Ref.df	χ²	*p*
AS.Both cues	2.06	2.57	4.48	0.205	1.00	1.00	2.56	0.110
NAS.Both cues	1.00	1.00	0.44	0.506	1.00	1.00	0.50	0.480
AS.No cue	2.09	2.61	9.78	**0.020**	1.00	1.00	7.99	**0.005**
NAS.No cue	1.00	1.00	12.66	< **0.001**	1.00	1.00	10.85	< **0.001**
Participants	47.68	646.00	153.93	< **0.001**	68.49	646.00	256.92	< **0.001**
	*R*^2^(adjusted) = 0.174 Deviance explained = 19.5%				*R*^2^(adjusted) = 0.170 Deviance explained = 18.2%			

GAMMs: generalised additive mixed models; SNR: signal-to-noise ratio; *SE*: standard error; AS: autistic group; NAS: non-autistic group, EDF: effective degrees of freedom.

Formula: Accuracy ~ Group*Cue + s(Trial, by = Group*Cue, *k* = 8) + s(Trial, bs = ‘fs’, *m* = 1). Significant *p*-values are presented in bold.

#### Parametric effects

In both models, the parametric coefficients revealed significant accuracy differences when comparing the baseline condition (both cues in the autistic group) to the other group–condition combinations. Specifically, accuracy was significantly lower in the no-cue condition for both groups. No significant group differences were observed in the both-cues condition, indicating comparable accuracy.

#### Time-varying effects (smooth terms)

Smooth terms of the models revealed significant non-linear changes in performance across trials, but only in the no-cue condition. Significant increase of accuracy was observed in both groups across both SNR levels, indicating improved performance over trials. In contrast, no significant trial effects were found in the both-cues condition for either group, reflecting their stable ceiling-level performance from the beginning of the task.

#### Group and condition contrasts over time (difference plots)

**Figure 5.**
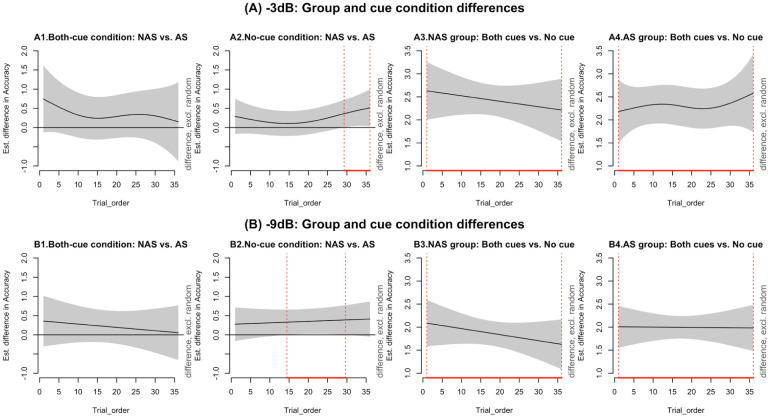
Estimated differences in accuracy over trials. The black line represents the estimated difference, with the grey shaded area indicating the 95% confidence interval. Red segments highlight trial ranges where the difference is statistically significant (*p* < 0.05).

To visualise when and where group and condition differences emerged during the task, we examined pairwise comparisons using difference plots (see Figure 5). These plots highlight time windows where significant contrasts appeared and are interpreted in light of the accuracy trends shown in Figure 4. In the no-cue condition, significant group differences emerged during the later trials (see Figure 5, A2 and B2), with non-autistic participants outperforming autistic participants from trials 29–36 at −3 dB and from trials 14–30 at −9 dB. As shown in Figure 4, this difference reflects the fact that accuracy in the no-cue condition continued to increase for the non-autistic group, while the autistic group’s performance remained more stable or variable. This widening gap suggests that the non-autistic group continued to improve with exposure, whereas the autistic group showed less consistent change.

Cue-related differences within each group were illustrated in Panels A3–A4 and B3–B4. For the non-autistic group (A3, B3), the difference between both-cues and no-cue conditions decreased over time, mirroring the upwards trend in no-cue accuracy seen in Figure 4. This suggests improved performance over trials. For the autistic group (A4, B4), the size of the cue-related difference remained relatively stable, especially at −3 dB. Figure 4 supports this and shows that while both-cues accuracy stayed high throughout, performance in the no-cue condition fluctuated and showed less overall improvement.

Taken together, both groups used the acoustic cues effectively when available. However, in the no-cue condition, only the non-autistic group showed steady gains over time. The autistic group also improved, but their performance was more variable, and they did not fully catch up in the later trials.

### Correlations

**Figure 6.**
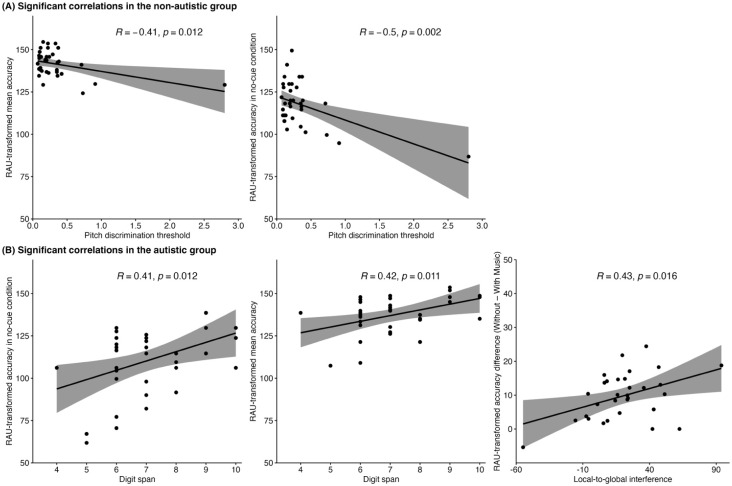
Significant correlations between accuracy and cognitive factors in the non-autistic group (A) and the autistic group (B). The grey shaded area indicated the 95% confidence interval across the mean. RAU: rationalised arcsine units.

Figure 6 presents significant correlations for both groups. In the non-autistic group, lower pitch discrimination thresholds (indicating better pitch processing) were associated with higher mean accuracy and better performance in the no-cue condition. A follow-up analysis excluding an outlier (> 3 *SD* from the mean) yielded consistent results (see Supplementary Material). In the autistic group, higher digit span scores were linked to better overall and no-cue accuracy. In addition, participants who showed stronger local-to-global interference, indicating weaker global processing, also showed larger accuracy declines in the presence of background music.

## Discussion

This study examined speech processing in autistic and non-autistic adults in a competing-speaker scenario with background music, which required selective attention to the target speaker in a dynamic auditory environment. As expected, autistic participants exhibited lower accuracy than their non-autistic counterparts, reflecting greater challenges in recognising target speech in noisy environments.

### Benefits of acoustic cues on mean accuracy

Both groups demonstrated higher accuracy and faster responses when at least one acoustic cue was present, highlighting the benefit of salient acoustic cues. This aligns with the study by Emmons et al. (2022), who reported improved performance in autistic participants when both gender and location cues were available, compared to one-cue conditions. However, while they instructed participants to attend to specific acoustic features before each trial, our participants had to detect and use speaker-related cues independently based on the callsign within the speech stream. This closely mirrors real-life listening, where explicit instructions are rarely available. Also, our task involved identifying information from sentences, rather than recalling isolated words, further encouraging ongoing cue integration. In addition, by incorporating a no-cue condition, our study extended the investigation to scenarios without salient cues, offering new insights into performance under more challenging conditions. Taken together, these features contribute to a more naturalistic assessment of SiN processing and demonstrate that autistic listeners can benefit from speaker-related acoustic cues even in tasks that more closely approximate everyday communication demands.

### Group differences in trial-level improvement

We expanded our analysis beyond mean accuracy to explore how performance changed over time across cue conditions using GAMMs. In the both-cues condition, both groups showed stable ceiling performance throughout the experiment. These findings suggest that autistic participants used acoustic cues as effectively as their non-autistic peers, indicating intact SiN processing in less demanding scenarios. However, in the more challenging no-cue condition, where two male speakers were collocated, both groups initially experienced processing difficulties but showed significant improvement over trials. Significant group differences emerged in later trials, with non-autistic participants achieving higher accuracy. Our results suggest that SiN performance is modulated by task complexity, and that autistic participants may exhibit disproportionate difficulties as auditory scene complexity increased (Bendo et al., 2024).

Two factors may explain this group difference. The first concerns reduced implicit learning and atypical auditory attention in autism. Although no explicit instructions were given regarding the target speaker’s identity or location, the same male speaker was consistently positioned at a fixed auditory location. Non-autistic participants may have implicitly detected this regularity (Reber, 1989), gradually becoming more familiar with the target voice and finding it easier to process over time (Holmes et al., 2021; Nygaard & Pisoni, 1998). In contrast, autistic participants may have struggled to form a stable auditory representation of the target speaker, potentially due to challenges with implicit learning (Lawson et al., 2018). This may relate to predictive coding accounts, which suggest reduced weighting of top-down predictions (Van de Cruys et al., 2014), potentially making it harder to develop expectations about the speaker’s voice or location. In addition, autistic individuals often exhibit atypical auditory attention, particularly under high-demand conditions (Čeponienė et al., 2003; Emmons et al., 2022; Keehn et al., 2013). Without salient cues to guide attention, they may have found it harder to consistently focus on the target stream. However, as implicit learning and attention were not directly measured, these interpretations require further investigation.

The second factor concerns group differences in processing strategies. Despite the lack of salient cues, non-autistic participants may have used vocal differences between speakers to segregate speech. Supporting this, better pitch discrimination was associated with higher accuracy in the non-autistic group, but not in the autistic group. While both groups showed similar non-vocal pitch discrimination ability, only non-autistic participants appeared to use this perceptual skill to support task performance. This indicates that autistic participants may have detected pitch differences but not spontaneously used them to guide stream segregation, possibly reflecting atypical top-down processing, as proposed by the predictive coding theory (Van de Cruys et al., 2014). However, it is also important to consider that although non-vocal pitch discrimination was comparable between groups, we did not assess vocal pitch perception, which tends to differ in autistic individuals and has been associated with variations in their SiN processing (Schelinski & Von Kriegstein, 2020). Autistic individuals may experience greater difficulty prioritising socially relevant acoustic cues, particularly when these cues are subtle or ambiguous (Hernandez et al., 2020; Schwartz et al., 2020).

Instead, autistic participants appeared to rely more on working memory to manage the increasing demands of the no-cue condition. Significant positive correlations between working memory and accuracy suggest that they engaged higher-level cognitive resources as a complementary strategy during SiN recognition. This aligns with research highlighting the role of working memory in mitigating SiN difficulties (Dryden et al., 2017). Such reliance may reflect broader differences in auditory processing. According to WCC theory (Happé & Frith, 2006), autistic individuals may focus on local acoustic details at the expense of integrating information into a coherent global representation. Difficulties in binding subtle acoustic cues (e.g. pitch) into a unified auditory object may have increased the cognitive demands of the task, thereby prompting greater working memory involvement. The additional load of maintaining task-relevant information in memory may also have contributed to the group differences in performance (Lau et al., 2023).

### The effect of background music

This study is the first to examine the effect of music on speech recognition in autism. While music reduced accuracy in both groups overall, a significant group difference emerged in the both-cues condition: the presence of music reduced accuracy in the non-autistic group, but not in the autistic group. With both cues available, non-autistic participants may have relied on automatic processing, which requires minimal attention effort (Schneider & Shiffrin, 1977). While efficient, such processing is more susceptible to unexpected distractions like background music. Thus, the decline in accuracy may not reflect the music’s inherent distractibility, but the heightened sensitivity of ceiling-level performance to even subtle increases in task demands. In contrast, autistic participants showed no reduction in accuracy, which may suggest less reliance on automatic processing even when both cues were available. Instead, they may have sustained a more effortful, controlled focus on the speech signals, which made their performance less influenced by the presence of music (Xu et al., 2024). These results challenge our initial hypothesis that autistic participants would be more vulnerable to background music due to their heightened interest in music over speech. The structured and instrumental nature of the music used in this study may have lacked the personal or social relevance needed to elicit heightened distraction (Kiss & Linnell, 2021; Nadon et al., 2021).

Correlation analyses revealed that cognitive processing styles influenced autistic participants’ susceptibility to background music. Autistic participants with stronger local biases exhibited greater performance declines in the presence of music. This was measured using the local-to-global interference score, which reflects difficulty in focusing on global patterns when conflicting local details are present. These results provide support for WCC theory (Happé & Frith, 2006). During SiN processing, a local bias may hinder the ability to group auditory elements into meaningful streams, making it more difficult to separate target speech from background music. As a result, background music may be processed as a distracting competing source, leading to greater interference and reduced performance. Event-related potential (ERP) studies support this interpretation, showing that while autistic individuals process individual acoustic elements accurately, they exhibit reduced neural responses when required to integrate multiple sound streams (Lepistö et al., 2009). This effect may have been especially pronounced in this study due to the use of a 0 dB SNR, which increased listening demands and likely intensified the impact of local processing bias.

### Limitations and directions for future studies

One limitation of this study relates to the nature of the stimuli, which may have reduced task demands and masked group differences. The use of predictable speech and emotionally neutral music likely made the task less challenging by minimising semantic and emotional interference. Future studies should use more naturalistic speech and varying music features (e.g. emotional tone, genre, lyrics) to better capture group differences under more realistic and cognitively demanding conditions (Brown & Bidelman, 2022; Russo & Pichora-Fuller, 2008; Shi & Law, 2010).

In addition, our conclusions are based solely on behavioural measures. Emerging evidence suggests that, despite similar accuracy, autistic individuals may show increased listening effort, reflected in greater pupil dilation (Xu et al., 2024) and reduced magnetoencephalography (MEG) responses (Fadeev et al., 2024). Future research should incorporate neurophysiological measures to provide a more comprehensive understanding of SiN processing in autism.

Finally, our sample consisted of verbally and cognitively able adults, which helped control for confounds but limited the generalisability to broader autistic populations. Moreover, although the sample size was based on a power analysis, the relatively small pilot sample used for estimation may have reduced the precision of those calculations. Larger and more diverse samples are needed to explore potential subgroup differences within the autism spectrum.

Despite these limitations, our findings suggest that autistic individuals can achieve comparable speech recognition performance when listening conditions are structured and low in distraction. This points to the potential value of technologies such as remote microphone systems (Schafer et al., 2014) or sound-field amplification (Wilson et al., 2021), which enhance the salience of target speech when background noise cannot be fully controlled. Moreover, the observed improvement in autistic participants’ performance over trials suggests that they may benefit from structured training. Since cue-based training has been shown to improve SiN perception in non-autistic individuals (Gohari et al., 2023), adapting similar interventions for autistic populations could enhance their ability to navigate multi-talker environments.

## Conclusion

This study highlights the role of acoustic cues and background music in SiN processing in autism. While autistic listeners faced general difficulties, they effectively used acoustic cues to support speech recognition and showed improvement with repeated exposure in the absence of cues, though their progress was slower than that of non-autistic participants. In addition, individual differences in sensitivity to background music highlight the heterogeneity of cognitive processing styles in autism, reinforcing the need for personalised support strategies.

## Supplemental Material

sj-docx-1-aut-10.1177_13623613251376484 – Supplemental material for Listening in a noisy world: The impact of acoustic cues and background music on speech perception in autism Supplemental material, sj-docx-1-aut-10.1177_13623613251376484 for Listening in a noisy world: The impact of acoustic cues and background music on speech perception in autism by Jiayin Li, Maleeha Sujawal, Zivile Bernotaite, Ian Cunnings and Fang Liu in Autism
