# Supplementary material for: Listening in a noisy world: The impact of acoustic cues and background music on speech perception in autism
Source: Autism. 2025 Oct 14;30(1):134–49. doi: 10.1177/13623613251376484 (PMC12717287; doi:10.1177/13623613251376484)
Supplement: sj-docx-1-aut-10.1177_13623613251376484 – Supplemental material for Listening in a noisy world: The impact of acoustic cues and background music on speech perception in autism [file sj-docx-1-aut-10.1177_13623613251376484.docx]

# Supplementary Material

## 1. Response screen

**Supplementary Figure 1**. Response screen used in the sentence identification task. The image of the dog (left) represents the target callsign that participants were instructed to attend to. Six colour-coded response panels (black, red, white, blue, green, pink) were displayed, each containing numbered response options (1–6, 8, 9). Participants responded by using the mouse to click on the square that matched the correct colour-number combination associated with the keyword spoken by the target speaker.


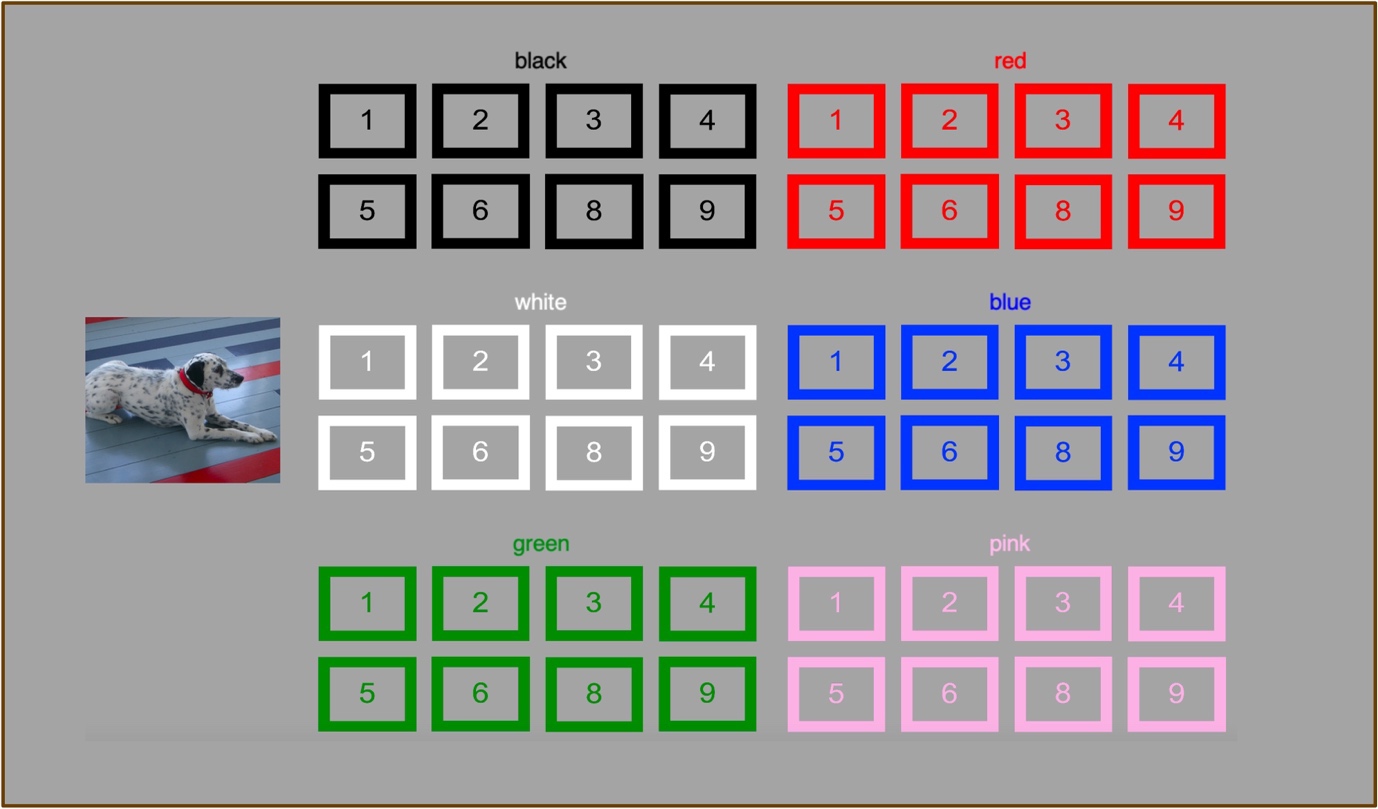


## 2. Power analysis

To determine the sample size, we conducted a power analysis using preliminary accuracy data (10 participants per group). Simulations were run using the mixedpower package (Kumle et al., 2021) with 1000 iterations, incorporating group, cue condition, background music, and their interactions as fixed effects.

To enhance statistical power, we grouped the location-cue and gender-cue conditions into a one-cue condition and applied Helmert coding to focus on two key contrasts: 1) no-cue vs. any cue conditions (the average of one-cue and both-cues); 2) one-cue vs. both-cues. This allowed us to prioritise the most relevant contrasts and enhance statistical power.

A generalised linear model (GLM) was fitted to pilot data, including fixed effects and random intercepts for participants and items, from which we obtained beta coefficients to define the smallest effect size of interest (SESOI). To account for uncertainty in effect size estimates, beta coefficients were reduced by 15% (Kumle et al., 2021).

As shown in Supplementary Figure 2, a sample size of 70 participants (n = 35 per group) would provide approximately 80% power to detect most effects of interest, particularly three-way interactions. Based on these results, we set our target sample size at 70 participants.

**Supplementary Figure 2**. Power analysis results. The plots show power estimates (y-axis) across different step sizes (x-axis) for main effects and interactions in the model. Each panel represents a specific effect or interaction, with error bars indicating uncertainty based on the smallest effect size of interest.


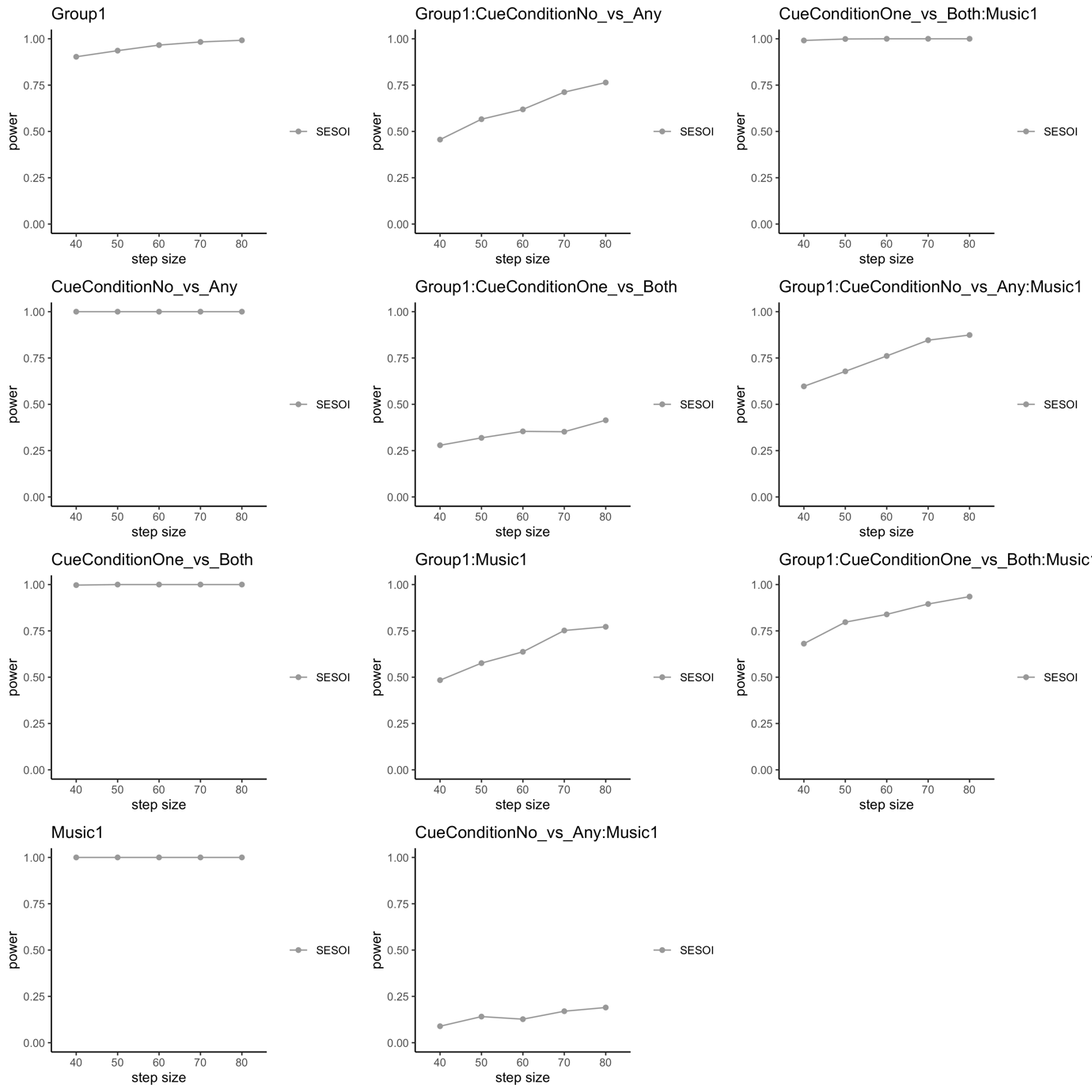


## 3. Pilot study

To determine the appropriate signal-to-noise ratio (SNR) for the distractor speech, a pilot study was conducted with three neurotypical, English-speaking participants with normal hearing. Participants listened to target speech while distractor speech was presented at four SNR levels: −3 dB, −6 dB, −9 dB, and −12 dB. An SNR of −3 dB means the distractor speech is 3 dB louder than the target speech, with progressively lower SNR levels indicating more challenging listening conditions.

The pilot study comprised 192 trials (48 per SNR level) across four blocks, with all four cue conditions randomly presented within each block. Results (see Supplementary Table 1) showed high accuracy at −3 dB and −6 dB (~80% overall, with near-ceiling performance in the both-cues condition). At −9 dB, accuracy declined to 60-70%, balancing task difficulty with reasonable performance. At −12 dB, accuracy dropped to ~50%, indicating substantial difficulty and possible reliance on guesswork.

Based on these findings, −9 dB was initially selected as it provided a balance between task difficulty and performance. However, given potential sensitivity to high sound levels in autistic participants (Danesh et al., 2021; Khalfa et al., 2004), the final experiment included a mix of −3 dB and −9 dB trials to reduce difficulty while maintaining ecological validity.

Notably, the effect of SNR level was not a focus of the study and was not analysed as an experimental variable. Instead, the inclusion of both levels served to introduce natural variation in task difficulty, support sustained participant engagement, and ensure the task was suitable for individuals with differing sensory profiles. During the experiment, the two SNR levels were randomly intermixed within blocks rather than presented in separate blocks. This design choice was intended to minimise learning or strategy effects that could arise if participants became aware of predictable shifts in difficulty. Randomisation across trials helped to maintain a more naturalistic and unpredictable listening environment, reflecting real-world situations in which background noise levels vary unexpectedly. Participants were not informed of the SNR changes in advance, further encouraging sustained attention and adaptive listening strategies. In summary, our approach prioritised participant accessibility, ecological validity, and engagement, while avoiding potential confounds related to sensory sensitivity and ensuring that the task remained feasible and realistic across both groups.

**Supplementary Table 1.** Mean accuracy rate of SNR levels included in the pilot study.

| SNR Level | −3 dB  Mean (SD) | −6 dB  Mean (SD) | −9 dB  Mean (SD) | −12 dB  Mean (SD) |
| --- | --- | --- | --- | --- |
| No Cue | 58% (50%) | 72% (45%) | 58% (50%) | 47% (51%) |
| Gender Cue | 86% (35%) | 78% (42%) | 72% (45%) | 50% (51%) |
| Location Cue | 85% (37%) | 86% (35%) | 72% (45%) | 58% (50%) |
| Both Cues | 96% (17%) | 97% (17%) | 66.7% (48%) | 72% (45%) |
| Grand average | 81% (39%) | 83% (37%) | 67% (47%) | 57% (50%) |

## 4. Music stimuli

The background music stimuli were derived from a validated set of film music excerpts developed by Eerola and Vuoskoski (2011). These excerpts were specifically selected to represent a range of emotional expressions, encompassing both discrete emotions—such as anger, fear, sadness, happiness, and tenderness—and positions along three bipolar dimensions: valence (positive–negative), energy arousal (high–low), and tension arousal (high–low). The selection process involved expert musicologists who curated unfamiliar film soundtrack segments to minimise the influence of participants’ prior associations. For our study, we selected excerpts from the “tender” category, which are characterised by high valence and low arousal, and were designed to evoke a peaceful, gentle emotional tone. These pieces are typically instrumental (without lyrics), feature slow tempo, smooth dynamics, and soft timbres, such as piano and strings. This selection was intended to mimic real-world ambient music while avoiding strongly emotional or arousing content that could greatly influence cognitive or emotional responses. To avoid perceptual discontinuities and ensure smooth transitions, we selected each music excerpt from the middle section of the original track, avoiding the beginning and end where dynamic or structural changes are more likely to occur. This approach ensured that all clips maintained consistent volume and texture throughout without the need for artificial fade-ins or fade-outs.

## 5. Linear mixed-effect models for accuracy

**5.1 Follow-up analyses of significant three-way interactions**

**Interaction between group, music, and cue1 (no-cue vs. any cues).** We first examined the group-by-cue1 interaction in trials with and without background music (*α* = 0.025). There was no significant interaction between cue1 and group in both trials with music, *χ^2^*(1) = 2.60, *p* = 0.107, OR = 0.76, 95% CI = [0.55, 1.05]; and trials without background music, *χ^2^*(1) = 0.83, *p* = 0.363, OR = 1.18, 95% CI = [0.83, 1.68]. Next, we investigated the music-by-cue1 effect within each group. The interaction between music and cue1 was not significant for either the non-autistic group, *χ^2^*(1) = 0.01, *p* = 0.933, OR = 1.04, 95% CI = [0.45, 2.38], or the autistic group, *χ^2^*(1) = 2.04, *p* = 0.153, OR = 1.81, 95% CI = [0.82, 4.02].

**Interaction between group, music, and cue2 (one-cue vs. both-cues).** We then examined the group-by-cue2 interaction in trials with and without background music (*α* = 0.025). There was no significant interaction between cue2 and group in trials with music, *χ^2^*(1) = 0.03, *p* = 0.854, OR = 0.97, 95% CI = [0.67, 1.39]; or without music, *χ^2^*(1) = 1.00, *p* = 0.316, OR = 1.30, 95% CI = [0.80, 2.11]. Similarly, we investigated the music-by-cue2 effect within each group. The interaction between music and cue2 was significant for the non-autistic group, *χ^2^*(1) = 8.15, *p* = 0.004, OR = 0.18, 95% CI = [0.05, 0.60], but not for the autistic group, *χ^2^*(1) = 2.16, *p* = 0.142, OR = 0.43, 95% CI = [0.14, 1.30]. Following this, we examined the simple effect of background music within the non-autistic group for each cue2 condition (*α* = 0.0125). In the both-cues condition, accuracy was significantly lower in trials with background music compared to those without background music, *χ^2^*(1) = 23.64, *p* < 0.001. This effect was reflected in the odds ratio (OR = 11.78, 95% CI [4.08, 34.00]), indicating a markedly higher likelihood of correct responses in the absence of background music. However, in the one-cue condition, no significant music effect was observed, *χ^2^*(1) = 0.24, *p* = 0.623, OR = 1.14, 95% CI = [0.66, 2.01].

## 6. Generalised additive mixed models (GAMMs)

**6.1 Accuracy GAMM reported in main text (no-cue vs. both-cues)**

**6.1.1 Rationale for model construction**

The effect of background music was excluded from GAMM analyses because it was presented randomly across trials, focusing on its incidental influence on overall performance rather than trial-level dynamics.

To balance analytical focus with statistical reliability, our main GAMM analysis focused on the contrast between the both-cues and no-cue conditions. This decision was guided by both empirical and methodological considerations. First, the gender and location cue conditions showed accuracy patterns that were broadly similar to the both-cues condition, with performance typically ranging from 85–100% across trials for both groups (see Supplementary Figure 3). This similarity indicates that the one-cue conditions did not produce meaningfully different behavioural outcomes from the both-cues condition. Second, including all four cue conditions would have significantly increased the number of group–cue comparisons and model complexity, reducing statistical power and increasing the risk of overfitting. Focusing on the most theoretically and behaviourally distinct contrast (i.e., both cues vs. no cue) allowed us to preserve statistical precision and maximise interpretability.

Although the analysis of reaction times (RTs) for accurate responses was included in our linear mixed-effects models (LMMs), it was not employed in the main GAMM analysis due to limitations in interpretability. Unlike LMMs, which focus on estimating mean effects across conditions, GAMMs model changes over time and rely on consistent data density within each condition to estimate smooth terms reliably. In our dataset, reduced accuracy especially in the no-cue condition resulted in fewer correct trials and thus sparser RT data. This sparsity compromised the stability of the smooth estimates. Moreover, because we examined RTs only for correct responses, more difficult conditions yielded RT data from a restricted and potentially non-representative subset of participants, introducing selection bias. Together, these limitations made RTs unsuitable for reliable GAMM modelling in the main analysis. In contrast, accuracy was recorded on every trial, providing a complete and more representative basis for modelling trial-level dynamics.

In response to reviewer suggestions, we included exploratory GAMM analyses using RT data as well as a four-condition accuracy model in the Supplementary Material. While these analyses provide a broader view of cue-specific and time-varying effects, their results should be interpreted with caution due to the limitations outlined above.

To avoid conflating the effects of cue condition and signal-to-noise ratio (SNR), we fitted separate models for each SNR level (−3 dB and −9 dB). This allowed for clearer interpretation of condition and group effects without confounding influences from SNR differences.

**Supplementary Figure 3**. Trial-level mean accuracy in each condition for autistic (AS) and non-autistic participants (NAS).


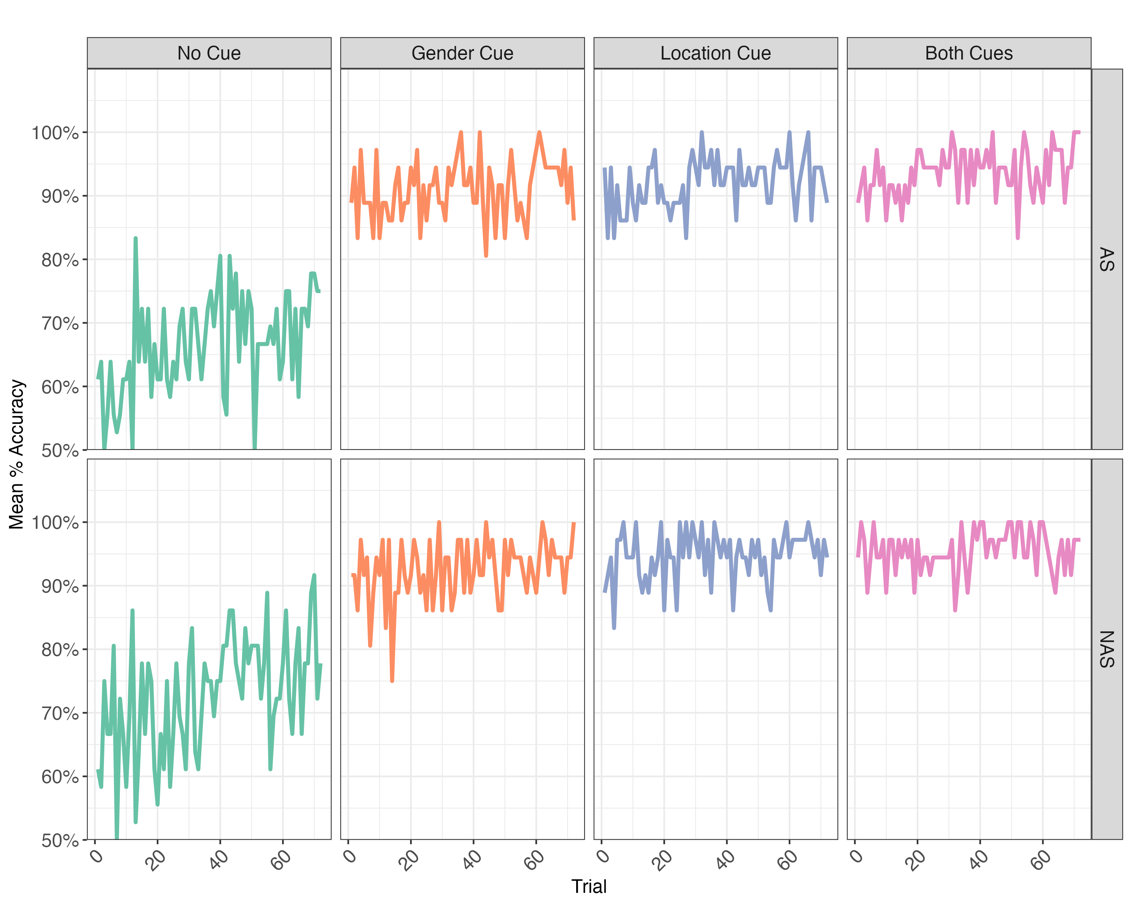


**6.1.2 Procedure of model fitting**

We constructed a series of nested models with increasing complexity and compared them using the compareML function from the *itsadug* package (van Rij et al., 2019). Three models were fitted:

- **Model 1 (m1)** included cue condition as a fixed parametric effect, a global smooth term for trial order to capture non-linear learning or adaptation effects, and a by-participant smooth to account for individual variability across trials.
- **Model 2 (m2)** extended m1 by allowing separate smooths for each cue condition, enabling trial-level effects to vary non-linearly across conditions.
- **Model 3 (m3)** replaced the fixed cue condition term with a Group × Cue interaction (two groups × two cue conditions). In m3, the autistic group in the both-cues condition served as the reference level, and separate smooths were estimated for each group–condition combination to capture potentially distinct learning curves.

Model comparisons were conducted sequentially (m1 vs. m2, then m2 vs. m3). Because compareML reports half of the likelihood-ratio statistic under “Difference,” the χ² values shown here correspond to those printed by the function, while the reported *p*-values are based on the doubled statistic (the conventional likelihood-ratio test). As likelihood-ratio tests for smooth terms provide approximate *p*-values, we interpreted them cautiously and placed emphasis on retaining theoretically motivated predictors and interactions. For transparency, we also report AIC differences and define ΔAIC as AIC(first model) − AIC(second model); thus, ΔAIC > 0 indicates the second model has the lower (better) AIC, whereas ΔAIC < 0 indicates the first model has the lower AIC. This approach keeps models aligned with our research questions even when purely statistical criteria suggest a simpler structure.

For the **−9 dB data**, model comparisons revealed that m2 significantly improved fit over m1, χ²(2) = 6.10, *p* = 0.002, supporting the inclusion of condition-specific trial order variability. The AIC difference was −1.48, slightly favouring the simpler m1, but we retained m2 because it captured condition-specific trial effects. Furthermore, m3 significantly improved fit over m2, χ²(6) = 6.95, *p* = 0.031, indicating that the Group × Cue interaction contributed significantly to explaining performance. The AIC difference was −3.89, favouring m2, but because testing this interaction was central to our research questions, we selected m3 as the best-fitting model for the −9 dB data.

For the **−3 dB data**, m2 provided a non-significant improvement over m1, χ²(2) = 0.69, *p* = 0.504. The AIC difference was +1.94, slightly favouring m2 over m1. Comparing m2 with m3 also yielded a non-significant result, χ²(6) = 3.72, *p* = 0.283, and the AIC difference (*ΔAIC =* −4.10) favoured the simpler m2. Taken together, there was no statistical or information-criterion evidence supporting the more complex models at this SNR. Nonetheless, we retained m3 as the final model to ensure consistency across SNR levels and to allow estimation of the theoretically important Group × Cue interaction.

To ensure that model complexity did not result in overfitting, we conducted extensive model criticism. Diagnostic plots were used to ensure key assumptions were met (Supplementary Figure 4). The Q-Q plots indicated that residuals closely followed a normal distribution, with only minor deviations in the tails, generally supporting the assumption of normally distributed residuals. The residuals vs. fitted values plots showed a scattered pattern without discernible structure, suggesting homoscedasticity and the absence of systematic trends in residuals. Temporal dependencies were assessed using the autocorrelation function (ACF) of residuals (van Rij et al., 2019). As shown in Supplementary Figure 4, ACF values at Lag 1 and beyond remained close to zero, with no significant deviations across all lags, indicating that the model adequately accounted for temporal dependencies. Therefore, no further corrections for autocorrelation were necessary.

**Supplementary Figure 4.** Accuracy model (No-cue vs. Both-cues) diagnostics for the selected GAMM for each SNR level.


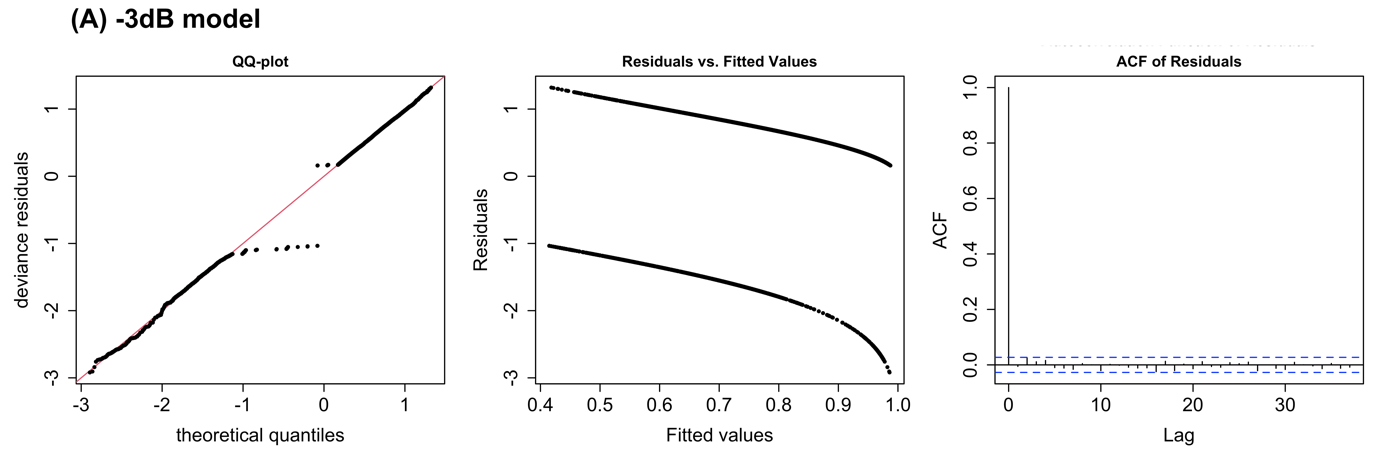


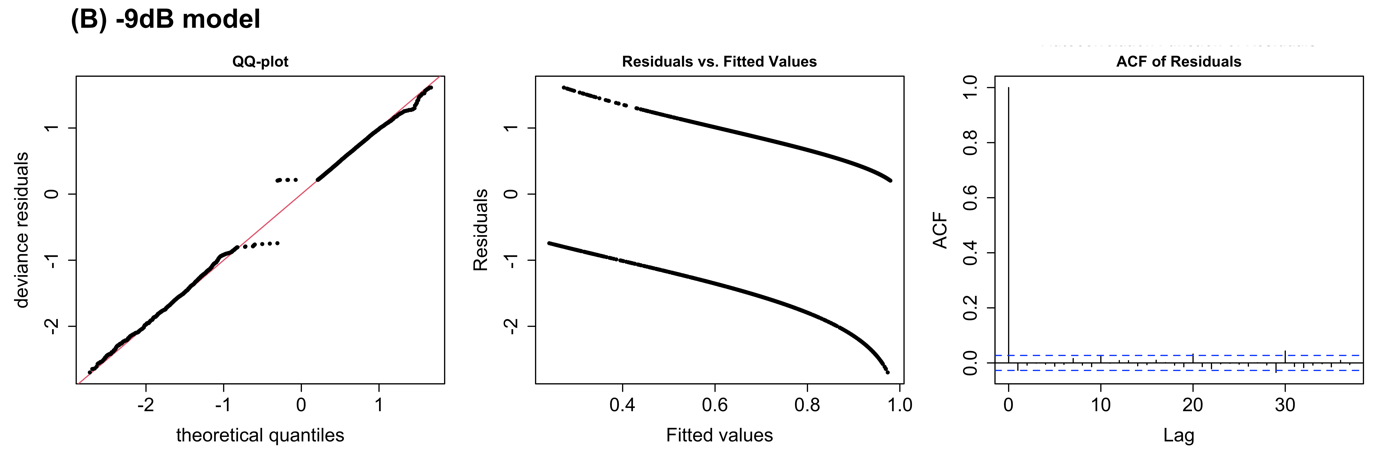


**6.2 Reaction time GAMMs (exploratory)**

**6.2.1 Procedure of model fitting**

In response to reviewer feedback, we conducted an exploratory GAMM analysis of reaction times (RTs) for correct responses, using a model-fitting procedure consistent with that applied to the accuracy data. Likelihood ratio tests were not applicable for the RTs’ GAMMs due to the way degrees of freedom are estimated in models with smooth terms. Specifically, generalised additive models use penalised smoothing, which can result in non-integer and even negative differences in effective degrees of freedom when comparing models. This occurs when more complex models include smooth terms that are heavily penalised so that they contribute little explanatory value. As a result, the assumption of nested models with valid, positive degrees of freedom required for likelihood ratio testing is violated. Accordingly, we based our model selection for RTs on AIC differences and theoretical interpretability.

For the −9 dB data, model 2 (m2), which included cue-specific smooths, showed a modest improvement in AIC (*ΔAIC = 1.66*) over the simpler global-smooth model (m1). Model 3 (m3), which included Group × Cue-specific smooths, significantly improved model fit relative to m2, and further reduced AIC (*ΔAIC = 4.39*). Based on these results, we selected m3 as the best-fitting and theoretically most appropriate model for the −9 dB reaction time data.

For the −3 dB data, m2 showed slightly worse fit than m1 (*ΔAIC = −0.26*), indicating that cue-specific smooths did not improve model performance. Model 3 (m3) further worsened fit compared to m2 (*ΔAIC* = −2.23). Accordingly, we selected m1 as the most parsimonious and best-fitting model for the −3 dB data. Notably, this model did not include the Group × Cue interaction of interest, as the data did not support the inclusion of more complex terms. We therefore report the −3 dB RT results for completeness but interpret them with caution, in contrast to the −9 dB analysis where model fit supported the inclusion of the interaction.

Model check was conducted for the RT-based GAMMs at both SNR levels (Supplementary Figure 5). The Q-Q plots showed that residuals were approximately normally distributed, with only mild deviations in the tails, consistent with expectations for reaction time data. Residuals vs. fitted values plots showed no discernible structure, suggesting no major violations of homoscedasticity. ACF plots revealed no notable autocorrelation, indicating that the models adequately accounted for temporal structure. These diagnostics support the appropriateness of the models for exploratory purposes.

**Supplementary Figure 5.** RTs model diagnostics for the selected GAMM for each SNR level.


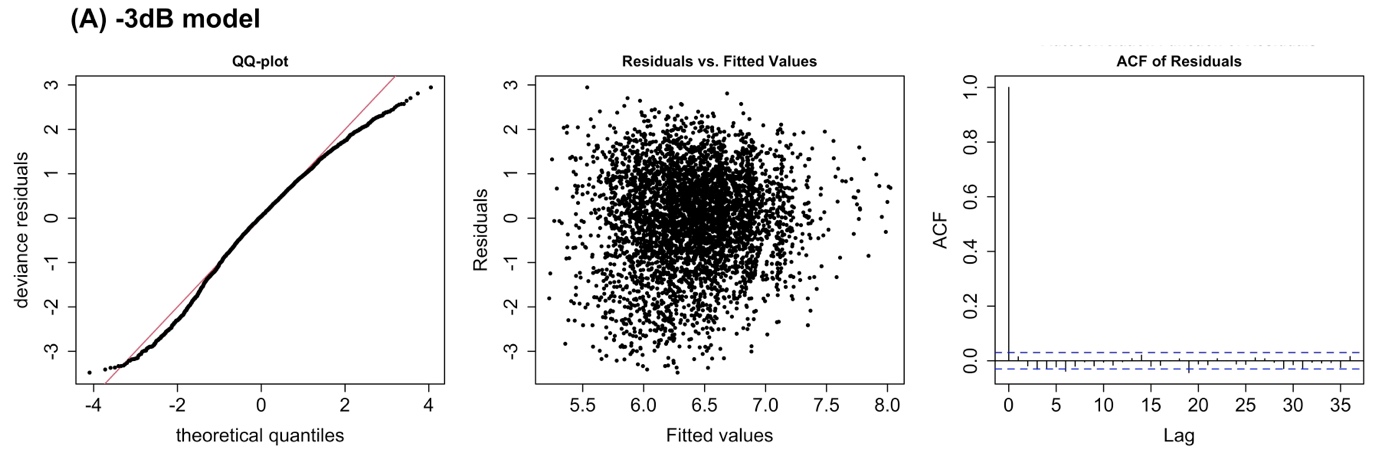


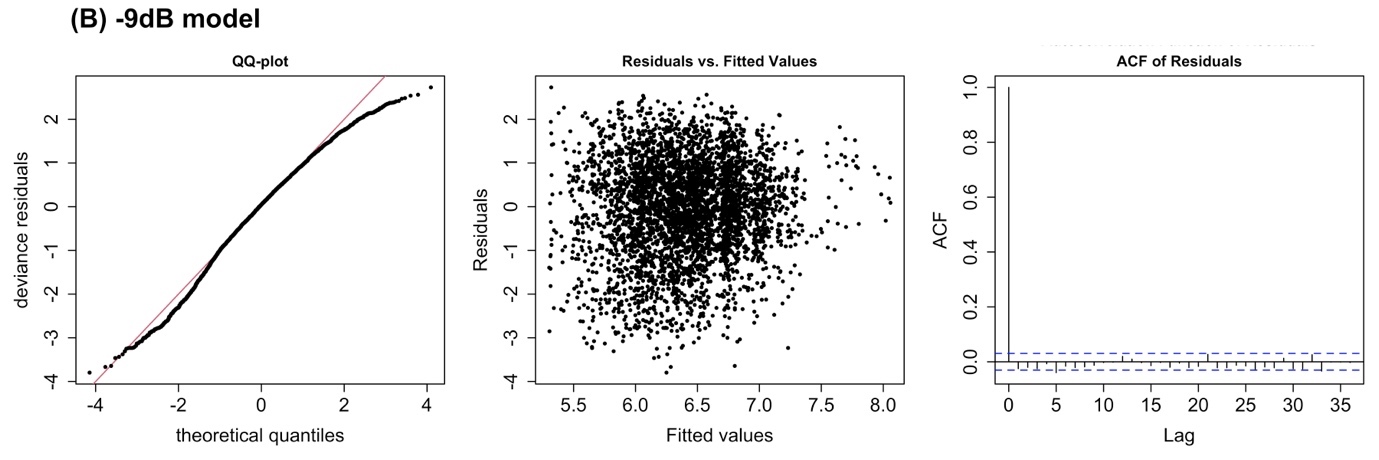


**6.2.2 Results**

For the −3 dB model, the parametric coefficients revealed a significant difference in RTs between the two cue conditions. RTs were significantly longer in the no-cue condition compared to the both-cues condition (*β* = 0.25, *p* < 0.001), suggesting that listeners responded more slowly when acoustic cues were absent. The smooth term for trial order was not statistically significant (*edf* = 1.00, *Ref.df* = 1.00, *F* = 2.45, *p* = 0.118), indicating no consistent change in RTs across trials when averaged across all participants and conditions. This likely reflects the limitations of the simpler model structure selected for the −3 dB condition, which did not include cue- or group-specific smooths due to concerns about overfitting. Consequently, the model could not capture potential trial-wise differences across conditions or groups. The final model explained approximately 24.2% of the deviance (*adjusted R²* = 0.263).

For the −9 dB model, the parametric coefficients revealed significant differences in RTs relative to the baseline (autistic group, both-cues condition). RTs were significantly longer in the no-cue condition for both the autistic group (*β* = 0.35, *p* < 0.001) and the non-autistic group (*β* = 0.20, *p* = 0.042). These findings align with the −3 dB results and further suggest that both groups responded more slowly when acoustic cues were absent. There was no significant RT difference between groups in the both-cues condition (*β* = –0.05, *p* = 0.635), indicating comparable response latencies when both cues were available. Analysis of the smooth terms revealed a significant decrease in RTs over trials only in the autistic group’s both-cues condition (*edf* = 1.00, *Ref.df* = 1.00, *F* = 3.90, *p* = 0.048). The final model explained approximately 26% of the deviance (*adjusted R²* = 0.282). Pairwise comparisons were examined using difference plots to evaluate trial-level effects of cue condition and group (Supplementary Figure 6). No significant group differences were observed in either the both-cues or no-cue condition, indicating comparable RTs between autistic and non-autistic participants within each cue condition. As expected, RTs were consistently faster in the both-cues condition than in the no-cue condition for both groups. This difference remained stable over trials for the non-autistic group, but the autistic group showed a gradual reduction in cue-related RT differences over time. Descriptive trend plots (Supplementary Figure 7) suggest this may reflect a decrease in RTs across trials in the no-cue condition for autistic participants, potentially indicating adaptation or improved tracking. However, due to reduced and uneven trial counts, these results should be interpreted with caution.

**Supplementary Figure 6.** Estimated differences in RTs over trials. The black line represents the estimated difference, with the grey shaded area indicating the 95% confidence interval. Red segments highlight trial ranges where the difference is statistically significant (*p* < 0.05).


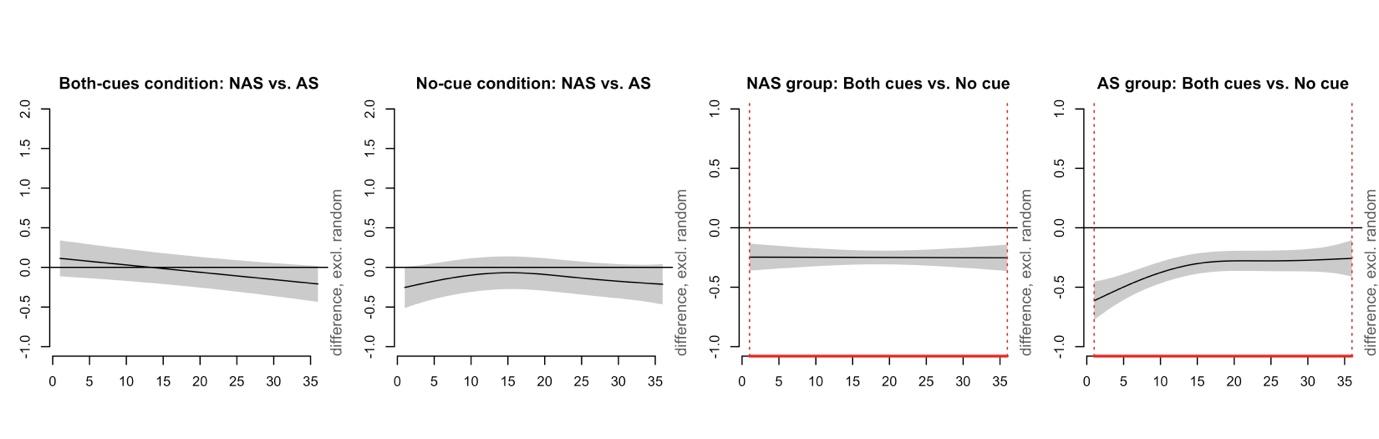


**Supplementary Figure 7**. The trend of mean RT changes across trial bins (every 6 trials) for different SNR levels across group and condition with the shaded area indicating the 95% confidence interval.


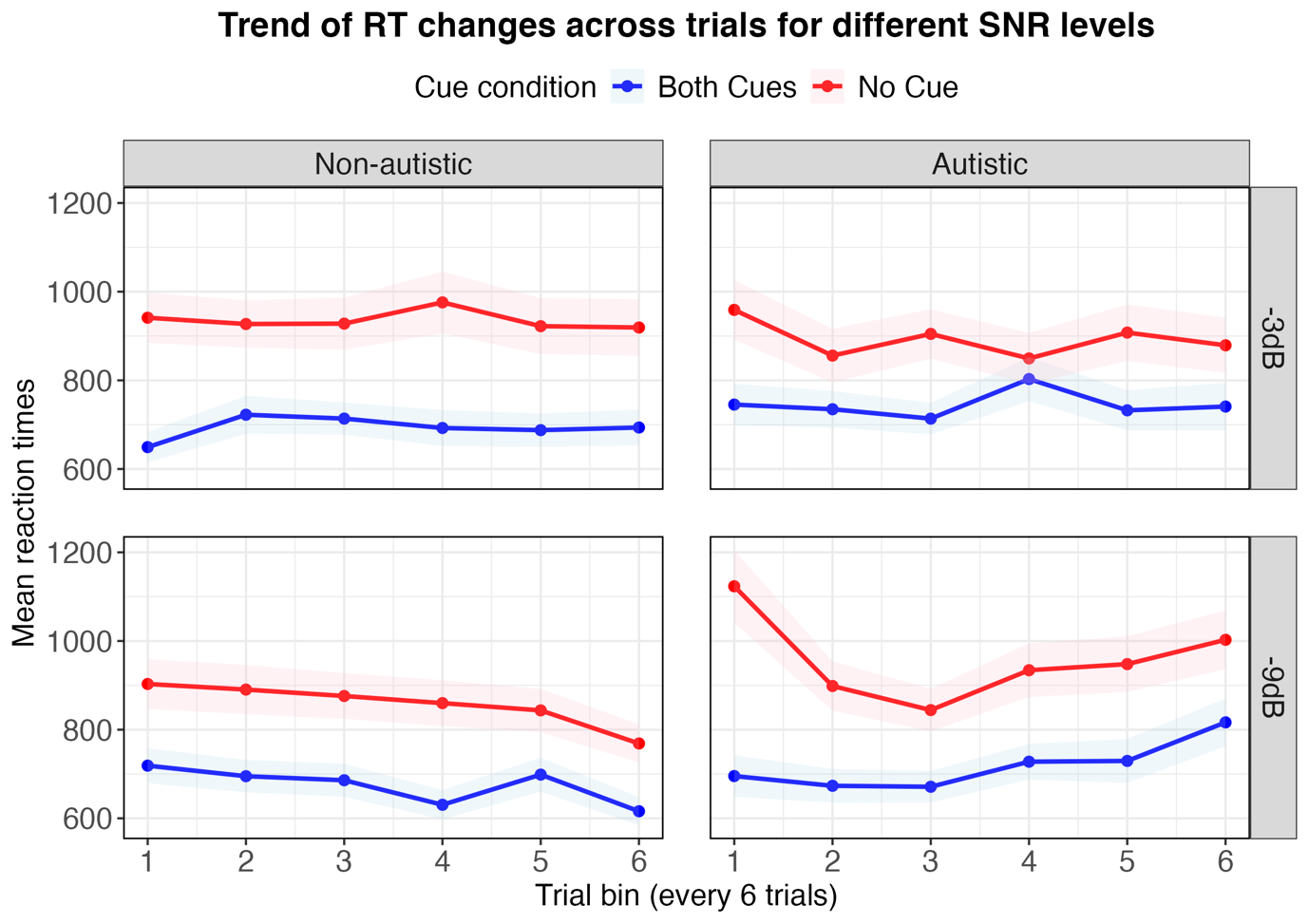


**6.3 Full accuracy-based GAMMs: all cue conditions (exploratory)**

As outlined earlier, our main analysis focused on the contrast between the no-cue and both-cues conditions due to its direct relevance to our research aims. In response to reviewer feedback, we conducted an exploratory GAMM analysis incorporating all four cue conditions (no cue, gender cue, location cue, both cues) to investigate potential cue-specific effects. We did not include pairwise difference plots in the four-cue model to avoid inflating Type I error. Given the large number of potential comparisons and the absence of inherent correction for multiplicity, such plots could lead to false positives. We therefore focused on interpreting parametric coefficients and smooth terms from the model summary.

**6.3.1 Procedure of model fitting**

The same model-fitting procedure described earlier was used, with the smoothing parameter k increased from 8 to 10 to better capture trial-level variability.

For the −3 dB data, model comparisons showed that m2 fit better than m1 (*χ²*(6) = 5.25, *p* = 0.105), and m3 further improved fit over m2 (*χ²*(12) = 4.15, *p* = 0.529), though neither comparison reached conventional significance. Nonetheless, we retained m3 for consistency across SNR levels and to enable estimation of the Group × Cue interaction.

For the −9 dB data, m2 did not improve model fit over m1 (*χ²*(6) = 0.04, *p* = 1.000), but m3 significantly outperformed m2 (*χ²*(12) = 14.60, *p* = 0.004), justifying inclusion of the interaction terms. Thus, m3 was selected as the best-fitting model for the −9 dB data.

Model diagnostics indicated a good fit for both final models (see Supplementary Figure 8). Deviance residuals were approximately normally distributed with only minor deviations in the tails. Residuals vs. fitted plots showed curved patterns expected for bounded data, and autocorrelation checks revealed no temporal dependency, supporting model adequacy.

**Supplementary Figure 8.** Accuracy model (4 cues) diagnostics for the selected GAMM for each SNR level.


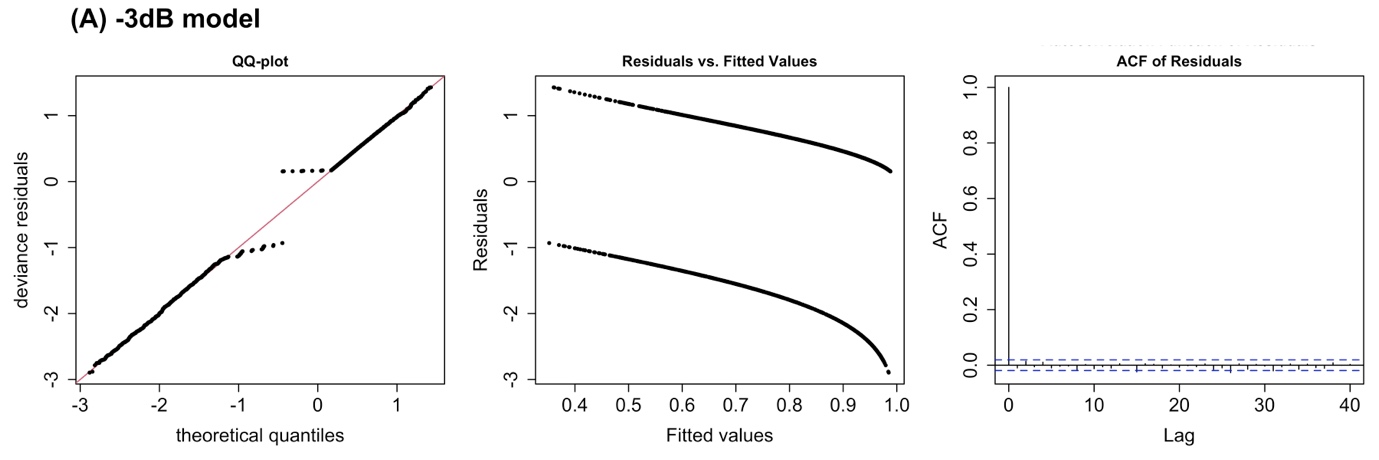


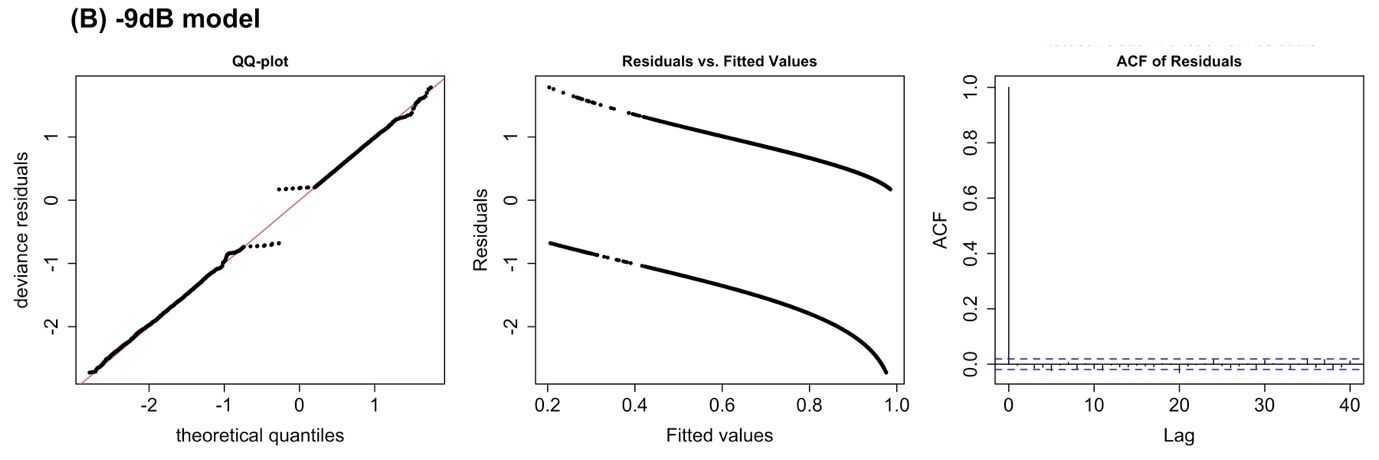


**6.3.2 Results**

At −3 dB, parametric results showed that the only group–cue combinations not significantly different from the baseline (autistic group, both-cues condition) were the non-autistic group in the both-cues and location-cue conditions. All other combinations showed significantly lower accuracy. Specifically, gender cue trials showed reduced accuracy for both the autistic group (*β* = −0.84, *p* < 0.001) and the non-autistic group (*β* = −0.70, *p* < 0.001). The location cue condition yielded a significant accuracy drop for the autistic group (*β* = −0.35, *p* = 0.047). No-cue trials showed the most pronounced reduction for both the autistic (*β* = −2.31, *p* < 0.001) and non-autistic (*β* = −2.07, *p* < 0.001) groups. Trial-level effects showed significant accuracy improvements in the no-cue condition for both groups (autistic: *edf* =2.10, *Ref.df* = 2.61, *χ^2^*= 9.73, *p* = 0.020; non-autistic: *edf* =1.00, *Ref.df* = 1.00, *χ^2^* = 12.61, *p* < 0.001), consistent with findings from the main analysis. The model explained 16.3% of the deviance (*adjusted R^2^* = 0.14). These findings reinforce our main analysis by showing that trial-wise improvements are primarily present in the no-cue condition.

At −9 dB, parametric effects indicated that accuracy was significantly lower only in the no-cue condition for both groups (autistic: *β* = −2.04, *p* < 0.001; non-autistic: *β* = −1.70, *p* < 0.001). All other group–cue combinations showed no significant difference from the baseline. For time-varying effects, significant improvements in accuracy over trials were found in the no-cue condition for both groups (autistic: *edf* =1.00, *Ref.df* = 1.00, *χ^2^* = 8.03, *p* = 0.005; non-autistic: *edf* =1.00, *Ref.df* = 1.00, *χ^2^*=10.81, *p* = 0.001). Additionally, the autistic group showed significant improvement in the gender cue condition (*edf* = 1.69, *Ref.df* = 2.10, *χ^2^* =7.54, *p* = 0.026), and a marginal effect was observed for the non-autistic group (*edf* = 1.26, *Ref.df* = 1.48, *χ^2^* = 5.54, *p* = 0.054). The model accounted for 18.2% of the deviance (*adjusted R^2^* = 0.16).

Together, these exploratory GAMMs broadly aligned with the main analysis, confirming that trial-level accuracy improvements were most consistent in the no-cue condition. At −9 dB, however, an additional improvement was observed in the gender cue condition for the autistic group (and marginally in the non-autistic group), suggesting some adaptation to gender cue information. But no such effect was observed at −3 dB. Descriptive trend plots are shown in Supplementary Figure 9.

**Supplementary Figure 9**. The trend of mean accuracy changes across trial bins (every 6 trials) for different SNR levels across group and condition (4 cues) with the shaded area indicating the 95% confidence interval.


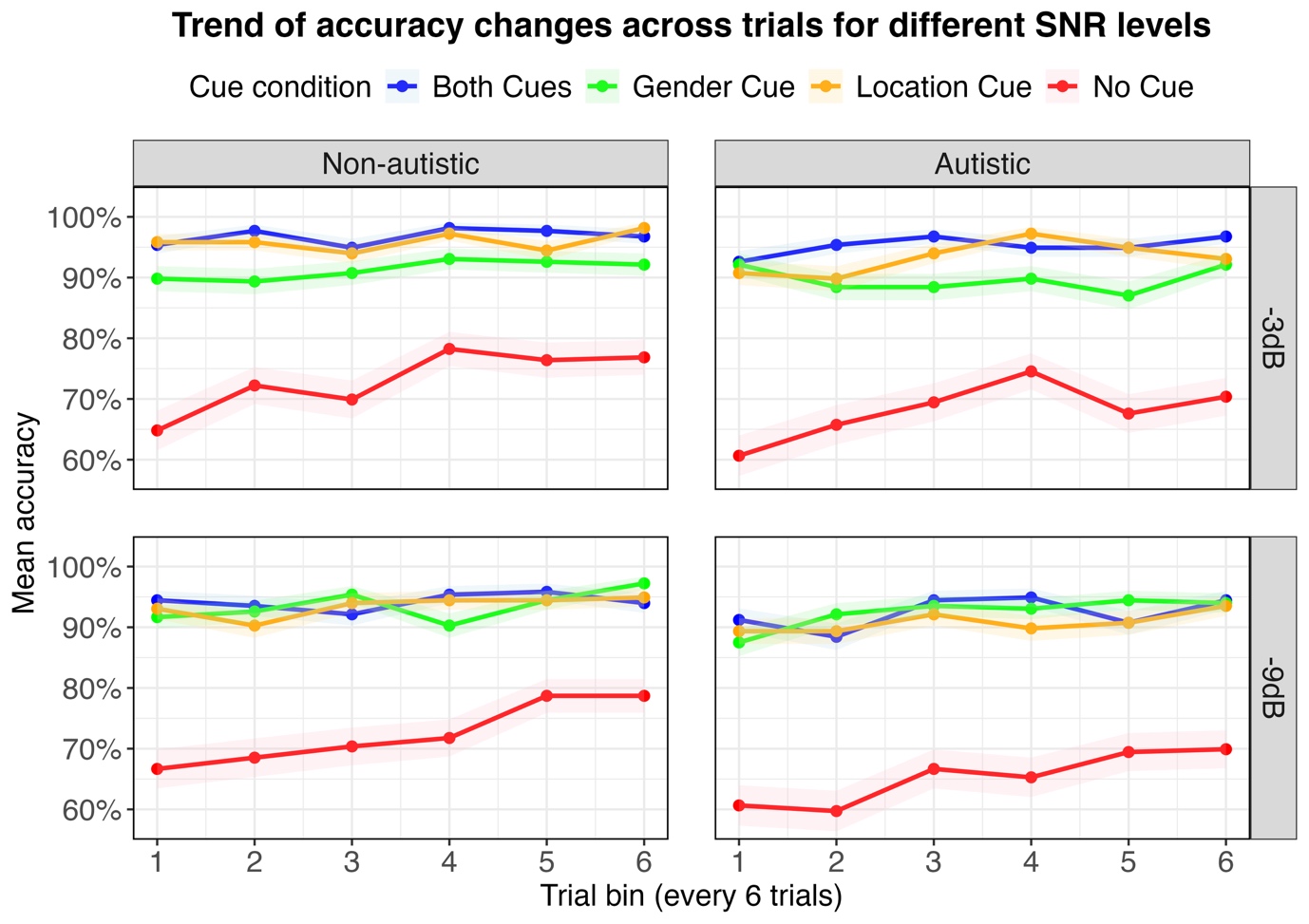


## 7. Correlations

To account for an outlier in pitch discrimination thresholds (>3 SD from the mean), we conducted a secondary analysis (see Supplementary Figure 10). After removing the outlier, better pitch processing remained significantly associated with higher accuracy in the no-cue condition for the non-autistic group.

**Supplementary Figure 10.** Correlations between pitch discrimination threshold and performance in the non-autistic group without the outlier. Accuracy values were transformed using the rationalised arcsine unit (RAU) transformation to normalise proportion data and reduce variance instability. The grey shaded area indicates the 95% confidence interval across the mean.


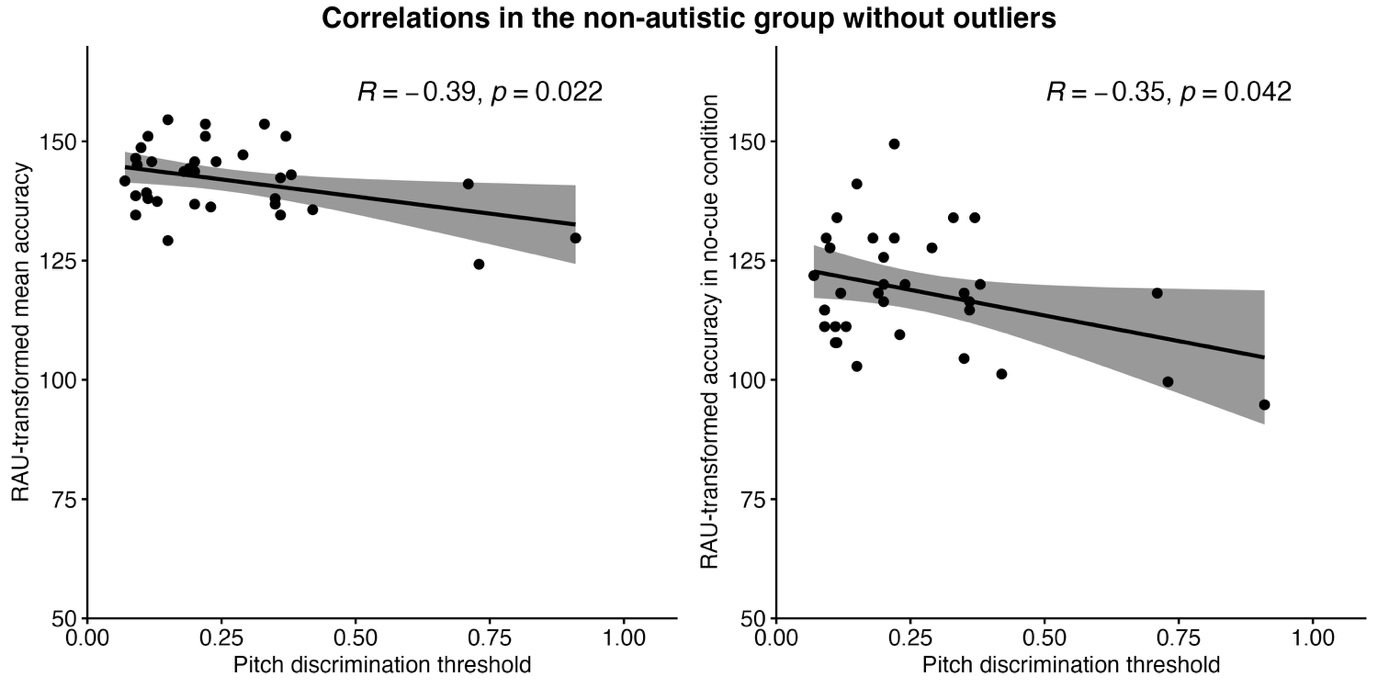


**References**

Danesh, A. A., Howery, S., Aazh, H., Kaf, W., & Eshraghi, A. A. (2021). Hyperacusis in Autism Spectrum Disorders. *Audiology Research*, *11*(4), Article 4. https://doi.org/10.3390/audiolres11040049

Eerola, T., & Vuoskoski, J. K. (2011). A comparison of the discrete and dimensional models of emotion in music. *Psychology of Music*, *39*(1), 18–49. https://doi.org/10.1177/0305735610362821

Khalfa, S., Bruneau, N., Rogé, B., Georgieff, N., Veuillet, E., Adrien, J.-L., Barthélémy, C., & Collet, L. (2004). Increased perception of loudness in autism. *Hearing Research*, *198*(1), 87–92. https://doi.org/10.1016/j.heares.2004.07.006

Kumle, L., Võ, M. L.-H., & Draschkow, D. (2021). Estimating power in (generalized) linear mixed models: An open introduction and tutorial in R. *Behavior Research Methods*, *53*(6), 2528–2543. https://doi.org/10.3758/s13428-021-01546-0

Van Rij, J., Hendriks, P., Van Rijn, H., Baayen, R. H., & Wood, S. N. (2019). Analyzing the Time Course of Pupillometric Data. *Trends in Hearing*, *23*, 2331216519832483. https://doi.org/10.1177/2331216519832483
